# Individual differences do not mask effects of unconscious processing

**DOI:** 10.3758/s13423-025-02679-5

**Published:** 2025-03-24

**Authors:** Itay Yaron, Nathan Faivre, Liad Mudrik, Matan Mazor

**Affiliations:** 1 Sagol School of Neuroscience, Tel Aviv University, Haim Levanon 55, Tel Aviv, Israel; 2 University Grenoble Alpes, University Savoie Mont Blanc, CNRS, LPNC, Grenoble, France; 3 School of Psychological Sciences, Tel Aviv University, Tel Aviv, Israel; 4 Department of Psychological Sciences, Birkbeck, University of London, London, UK; 5 Wellcome Centre for Human Neuroimaging, University College of London, London, UK; 6 All Souls College and Department of Experimental Psychology, University of Oxford, Oxford, UK

**Keywords:** Unconscious processing, Individual differences, Consciousness

## Abstract

A wave of criticisms and replication failures is currently challenging claims about the scope of unconscious perception and cognition. Such failures to find unconscious processing effects at the population level may reflect the absence of individual-level effects, or alternatively, the averaging out of individual-level effects with opposing signs. Importantly, only the first suggests that consciousness may be necessary for the tested process to take place. To arbitrate between these two possibilities, we tested previously collected data where unconscious processing effects were not found (26 effects from 470 participants), using five frequentist and Bayesian tests that are robust to individual differences in effect signs. By and large, we found no reliable evidence for unconscious effects being masked by individual differences. In contrast, when we examined 136 non-significant effects from other domains, two novel non-parametric tests did reveal effects that were hidden by opposing individual results, though as we show, some of them might be driven by design-related factors. Taken together, five analysis approaches provide strong evidence for the restricted nature of unconscious processing effects not only across participants, but also across different trials within individuals. We provide analysis code and best-practice recommendations for testing for non-directional effects.

**Supplementary information:**

The online version contains supplementary material available at 10.3758/s13423-025-02679-5.

## Introduction

Our brains simultaneously perform complex information processing functions and yet, at any given moment in time, only a small subset of these functions is accompanied by conscious experience. This raises the question: Which brain functions depend on consciousness, and which functions can take place without it?

One approach to investigating the scope and limits of unconscious processing is to measure the effect of different stimulus features on behaviour, while making sure that the stimulus itself is not consciously perceived (for review, see Kouider & Dehaene, 2007; Reingold & Merikle, 1988). If a stimulus feature affects behaviour even when the participant is not aware of the stimulus, being conscious of the stimulus cannot be necessary for processing that feature.

**Fig. 1:**
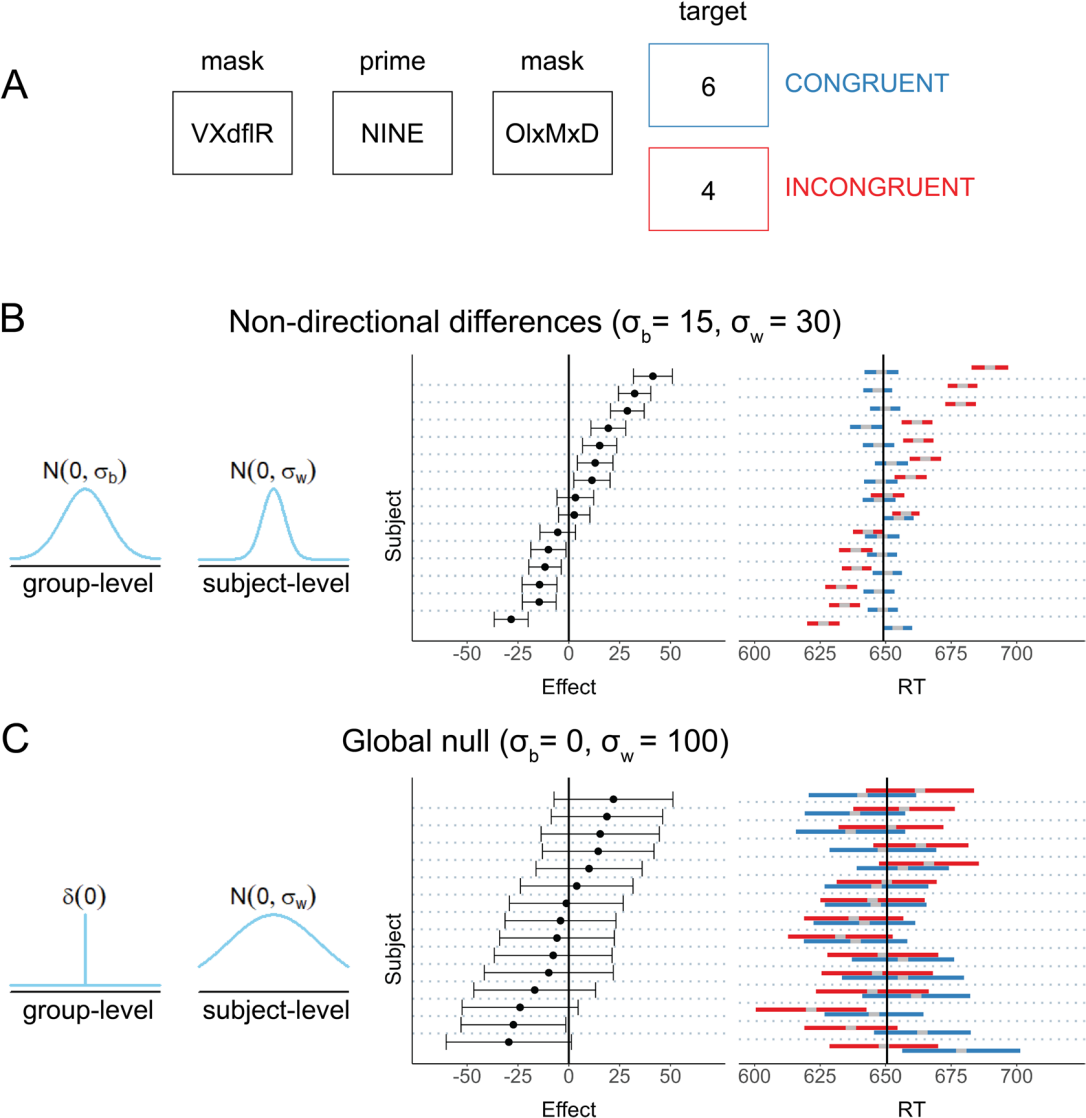
Simulated unconscious priming data. Simulated data demonstrating how true effects of unconscious priming can be masked by heterogeneity at the population level. **Panel A:** Stimuli in a typical unconscious processing experiment (based on Dehaene & colleagues, 1998). Participants make speeded decisions about a consciously perceived target stimulus (e.g., its magnitude: being larger or smaller than the number 5). The presentation of the target stimulus is preceded by a prime stimulus, which is masked from awareness. Decision time is measured as a function of prime-target agreement: congruent (blue) or incongruent (red). **Panels B** **and** **C: Left:** Simulation parameters controlling the within (${\sigma }_{w}$) and between (${\sigma }_{b}$) participant SD. **Right:** The results that were generated using the simulation parameters. Each point depicts the measured individual-level summary statistics for the difference between the mean reaction times (RTs) of each condition (congruent and incongruent) with 95% confidence interval ($C{I}_{95}$) around each difference estimate, and the blue and red segments depict the $C{I}_{95}$ around the average of RTs in each condition separately (the grey segment in the middle of each CI) in the congruent and incongruent conditions, respectively. A constant of 650 ms was added to the RTs in both panels for presentation purposes. **Panel B:** A *non-directional differences* scenario (simulated using the parameters ${\sigma }_{b}$= 15, ${\sigma }_{w}$= 30). **Panel C:** A *global null* scenario (no effect of the experimental manipulation; simulated using the parameters ${\sigma }_{b}$= 0, ${\sigma }_{w}$= 100). Since standard directional tests rely on individual-level summary statistics, they cannot arbitrate between the scenarios described in the two panels

For example, Dehaene and colleagues (1998) studied the role of consciousness in semantic processing. In their seminal study, they presented a number word stimulus (henceforth, the prime), followed and preceded by strings of random letters, acting as backward and forward masks, rendering it invisible. Then, a fully visible number stimulus was presented (henceforth, the target; see Fig. 1A). Participants were instructed to report whether the target stimulus was greater or smaller than the number five. Unconscious semantic priming was demonstrated by showing that participants responded faster in congruent trials, when the target and the prime were both smaller or larger than five (see again Fig. 1A). This suggests that the numerical magnitude of the prime was processed unconsciously, affecting the response to the visible target number (for critical assessment and discussions, see Damian, 2001, Naccache & Dehaene, 2001). In similar studies, participants were reported to unconsciously perform other high-level functions such as arithmetic operations (Sklar et al., 2012), extract and integrate word meanings (Damian, 2001; Sklar et al., 2012; Van Gaal et al., 2014), or scenes and objects (Mudrik et al., 2011), detect errors (Charles et al., 2013), and exert inhibition over responses (Van Gaal et al., 2008) to stimuli that were masked from awareness. Findings of high-level processing in the absence of consciousness served to inform and reform theories of consciousness (Dehaene & Naccache, 2001; Lamme, 2020; Lau & Rosenthal, 2011; Oizumi et al., 2014).

However, more recent work has called into question some of these previous findings and their interpretations. First, many of the original results do not replicate when tested in independent samples of participants (using direct replications, e.g., Biderman & Mudrik, 2018; Moors & Hesselmann, 2019; Stein et al., 2020, or conceptual replications, e.g., Hesselmann et al., 2015, 2016; Rabagliati et al., 2018). Second, some of these findings might be driven by residual consciousness in a subset of trials due to unreliable awareness measures (Meyen et al., 2022; Moors & Hesselmann, 2018; Rothkirch & Hesselmann, 2017; Shanks, 2017; Zerweck et al., 2021). Indeed, when re-analyzed to properly control for this possibility, some of these effects disappear (Meyen et al., 2022; Shanks, 2017). As a result, the scientific pendulum seems to be receding back to a narrower account of unconscious processing, consistent with a functional role of consciousness in most aspects of cognition (Balota, 1986; Meyen et al., 2022; Moors et al., 2017; Peters et al., 2017).

Overall, the field is still far from reaching a consensus regarding the scope and limits of unconscious processing. Although progress has been made in recent years toward improving methodology in unconscious processing studies, revealing the functional role of consciousness in cognition and perception remains difficult. Here we consider a largely neglected limitation of unconscious processing studies: By focusing on the average of signed (i.e., directional) single-participant summary statistics (e.g., subtraction of reaction times (RTs) between two conditions), previous investigations require not only that unconscious processing should leave a trace on behaviour, but also that this trace should be qualitatively similar across different participants (i.e., that the experimental manipulation would affect most participants in the same direction). We note that though this second requirement is intuitive, it is orthogonal with the theoretical question at stake; our main concern is whether a given stimulus feature can affect behaviour in the absence of consciousness, yet this does not necessarily imply that it affects all participants in the same way. This way, previous analyses of unconscious processing may have been too *conservative*, potentially missing effects that happen to vary between different participants (for a similar argument regarding cognitive science in general, see Ince et al., 2022).

On the face of it, pronounced individual differences in unconscious processing effects on cognition and perception seem possible, even likely. Indeed, using objective measures of awareness such as discrimination ability (direct tasks; Schmidt & Vorberg 2006; Schmidt & Biafora, 2024), previous research revealed heterogeneity in participants’ susceptibility to different masking paradigms, and in the effects of design choices on participants’ ability to consciously perceive the masked stimulus. Participants have been shown to reliably vary in their susceptibility to the attentional blink (Martens et al., 2006), and in the speed of breaking perceptual suppression – both in the breaking continuous flash suppression paradigm (b-CFS; Sklar et al., 2021) and in the breaking repeated mask suppression one (b-RMS; Abir & Hassin, 2020). Furthermore, manipulating stimulus onset asynchrony (SOA, Albrecht et al., 2010; see also Biafora & Schmidt, 2020) and mask contrast (Biafora & Schmidt, 2020) has been shown to produce different, sometimes opposite effects on metacontrast masking in different participants. Finally, visual imagery had different effects on the conscious perception of different participants in a binocular rivalry setting (Dijkstra et al., 2019). Some qualitative differences have been linked to variability in processing speed (Martens et al., 2006), genetics (Maksimov et al., 2013), and brain anatomy and physiology (Boy, Evans et al. 2010a; Van Gaal et al., 2011).

Critically, unconscious processing effects (i.e., indirect tasks; Schmidt & Vorberg, 2006) have also been shown to vary as a function of task parameters and across participants. At the heart of these findings are reports of “negative” priming effects: cases where the processing of the target was facilitated by an incongruent prime, rather than a congruent one. Indeed, masked priming effects changed in magnitude and even flipped in sign as a function of the interval between prime and target (Boy & Sumner, 2010; Boy & Sumner, 2014; Parkinson & Haggard, 2014; Schlaghecken & Eimer, 2004), or the presentation duration of stimuli rendered unconscious using either crowding (Faivre & Kouider, 2011) or CFS (Barbot & Kouider, 2012; Faivre et al., 2012). Negative effects were also observed for higher level features such as perceptual expectations: Bolger and colleagues (2019) showed that while most participants responded faster to upright faces in a b-CFS task, some responded faster to upside-down faces. Different hypotheses were laid out over the years regarding the driving mechanisms of the counter-intuitive negative priming effects. Among others, response inhibition of initial prime activations (Boy, Husain et al., 2020b; Eimer & Schlaghecken, 2003) or neural habituation (Barbot & Kouider, 2012; Faivre et al., 2012; Faivre & Kouider, 2011; Jacob et al., 2021) were suggested. Taken together, it is not clear if, and to what extent, unconscious effects are subject to meaningful individual variability. Crucially, if they are, then some previously reported null results might actually be true effects, masked by such variability.

The paper proceeds as follows. We first simulate a setting where a strong effect of unconscious processing on behaviour is entirely missed in standard analysis, due to pronounced inter-individual differences. We then show that the same effect is revealed when using three tests that are robust to population variability: the global null prevalence test (Donhauser et al., 2018), Bayesian hierarchical modelling (Haaf & Rouder, 2019), and a test based on analysis of variance (ANOVA; Miller & Schwarz, 2018). Importantly, unlike common measures of reliability which are used to directly estimate individual differences (see Parsons et al., 2019), the above tests do not quantify individual differences, but measure group effects in a way that is robust to such differences. Hence, they provide researchers with the appropriate tools for detecting unconscious effects even if pronounced individual differences exist, without depending on that being the case.

We then apply these tests to data gathered from eight unconscious processing studies (reporting 26 non-significant effects), and show that the same three tests support the null hypothesis according to which the behaviour of individual participants is unaffected by unconscious cognition and perception. This strengthens claims for a true absence of an effect in these studies. Finally, we propose two non-parametric alternatives that provide improved sensitivity and specificity, avoiding potentially unjustified statistical assumptions regarding the data-generating process. Our tests successfully reveal effects on multisensory integration, visual search, spatial attention and confidence ratings that could not be detected using standard directional analysis. However, similar to the three other approaches, our tests reveal no effects when applied to the studies of unconscious processing examined here. We conclude that existing data are most consistent with the absence of influences of unconscious stimuli on cognition and perception, not only at the population, but also at the single-participant level.

## Simulating non-directional unconscious effects

To provide a conceptual demonstration of how true causal effects of unconscious processing can be masked by inter-individual differences in effect signs, we simulated a typical experiment using a within-participants manipulation (Fig. 1). Specifically, we generated trial-by-trial data from a standard unconscious priming experiment. For each simulated participant, we generated RT data from two conditions (corresponding to congruent and incongruent primes in unconscious processing studies). Individual-level effect sizes (in milliseconds) were sampled from a normal distribution centred at zero (${e}_{i}\sim \mathcal{N}\left(0,{\sigma}_{b}\right)$, where ${e}_{i}$ denotes the true effect size of the ${i\text{th}}$ participant and ${\sigma }_{b}$ the between-participant standard deviation. Then, the trial-by-trial RTs of each participant and condition were generated according to each participant’s true effect score (${e}_{i}$), the relevant condition ($c\in \{\text{1,0}\}$, where $c=1$ denotes the incongruent condition, and $c=0$ denotes the congruent condition), and the within-participant standard deviation (${\sigma}_{w}$) ($R{T}_{i,c}\sim \mathcal{N}\left(0,{\sigma}_{w}\right)+c*{e}_{i}$).^1^

**Fig. 2:**
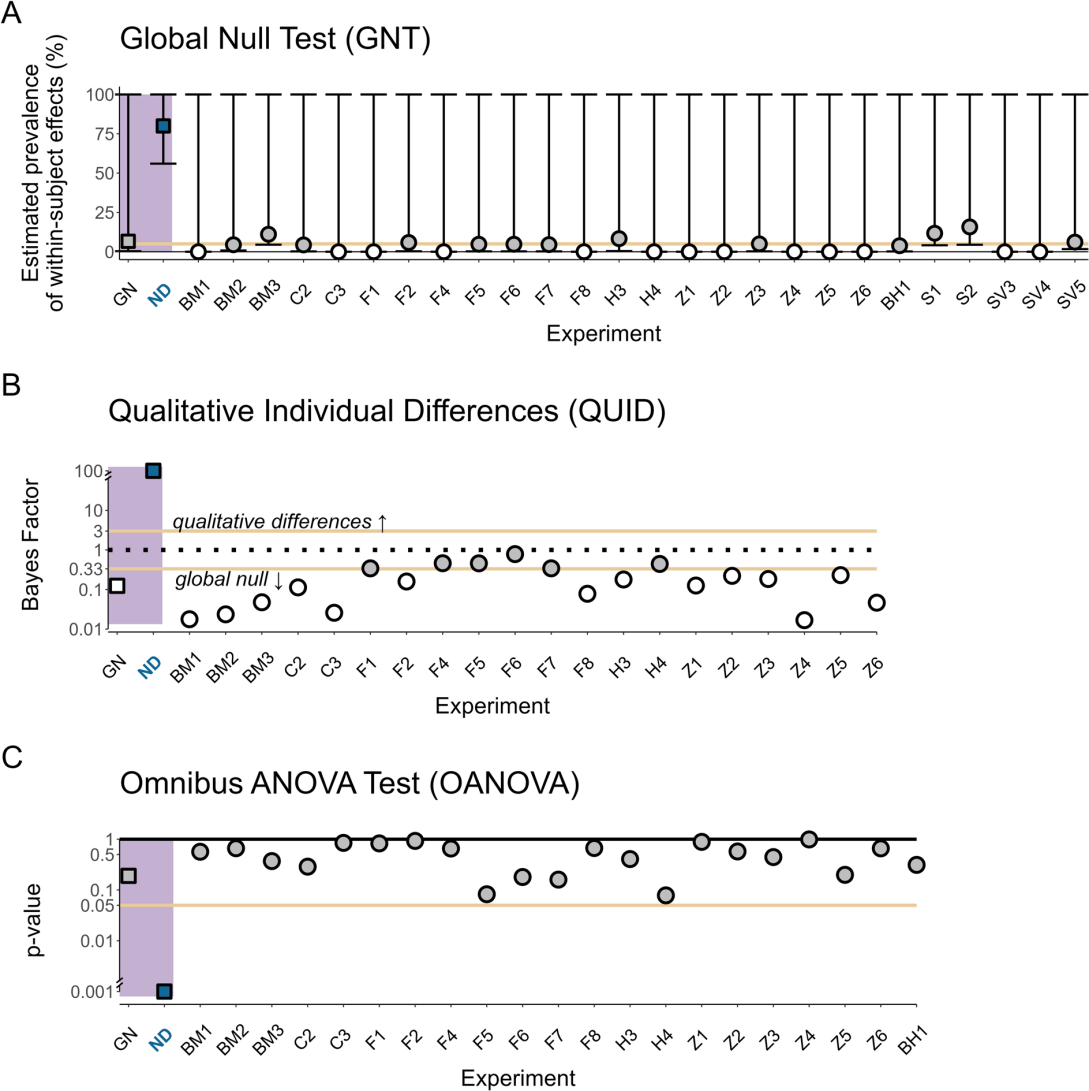
Reanalysis of unconscious processing effects. The results of applying the GNT (**A**), QUID (**B**), and OANOVA (**C**) tests to effects that produced null results in a non-parametric directional test and to simulated data (the Non-directional effect (ND) and Global Null (GN) simulations described above, presented as square-shaped markers). Effect labels appear on the x-axis. **Panel A:** The estimated prevalence of an unconscious effect in each of the cases, using GNT (Donhauser et al., 2018). Segments depict the one-sided 95% CI ($C{I}_{95}$) for the prevalence estimate, hence for GNT all CIs include 100% by definition, and the crucial test for an effect is whether the lower bound of each CI includes the $\alpha$ level used to test for individual-level effects (${\alpha}_{individual}$). The solid orange line indicates the expected prevalence of 5% significant individual-level effects, given that individual effects were tested using ${\alpha }_{individual}=0.05$ (type-1 error rate). **Panel B:** Bayes factors for the comparison between a random effects model that takes into account potential differences in effect signs and the global null model. White markers depict cases where moderate evidence for the global null model was found, while grey markers indicate inconclusive results. The dashed black line indicates a BF of 1 (no preference for either model), and the solid orange lines indicate a BF cutoff of 3. **Panel C:** The p-values obtained by the OANOVA test (Miller & Schwarz, 2018). Blue and grey markers indicate significant and non-significant results, respectively. For illustration purposes, BF and significance values are presented on a logarithmic scale on the y-axis

In two simulations, we manipulated two factors: the between-participant standard deviation (SD) over effect sizes (${\sigma }_{b}$), and the within-participant SD over RTs within each condition (${\sigma }_{w}$). This resulted in two distinct scenarios under this framework: (1) a *qualitative* or *non-directional differences* scenario, where the parameters of the generative model for all individuals were set to generate a true, non-zero effect (i.e., ${e}_{i}\ne 0$, for all individuals), but individual-level effects largely vary in magnitude and sign (${\sigma }_{b}$= 15, ${\sigma }_{w}$= 30; Fig. 1B), and (2) a *global null* scenario (Allefeld et al., 2016; Nichols et al., 2005), where no single participant is affected by the experimental manipulation (${\sigma }_{b}$= 0, ${\sigma }_{w}$= 100; Fig. 1C). We simulated ${N}_{t}$=100 trials per condition from ${N}_{p}$=15 participants per scenario, noting that the general principle holds for other sample sizes and number of trials.

First, we analyzed this simulated data using a two-sided paired t-test on the differences in mean RTs between the two conditions. This is the standard protocol for testing if unconscious processing took place. In both simulations, we obtained a null result, revealing no evidence for a difference in RT between the congruent and incongruent conditions (*non-directional differences*: $M=5.52$, 95% CI $\left[-5.49, 16.54\right]$, $t\left(14\right)=1.08$, $p=.300$; *global null*: $M=-2.78$, 95% CI $\left[-12.09, 6.53\right]$, $t\left(14\right)=-0.64$, $p=.532$). Importantly, in the *non-directional differences* simulation, all participants were affected by the experimental manipulation (that is, their true effect sizes were different from zero). Thus, this commonly used approach systematically misses true causal effects of the experimental manipulation whenever they are inconsistent between participants.

To reiterate, a standard t-test misses existing individual-level effects because, operating on individual-level summary statistics, it cannot differentiate between within-participant variability in the dependent variable (noise) and meaningful between-participant variability. In recent years, researchers sought to address this limitation, advocating for the use of *non-directional* statistical methods that incorporate both within and between-participant variability. Here, we examined three non-directional approaches, all capable of detecting effects that exist within at least a single participant, without relying on group-level assumptions about the consistency of effect signs across individuals. As a result, a non-directional finding indicates that an effect exists within single participants without committing to whether the average effect at the group-level is positive, negative, or null.

First, the *prevalence global null* approach (Donhauser et al., 2018; henceforth GNT) tests if the prevalence of individual-level effects in a given population (the proportion of individuals showing an effect) is greater than zero. The prevalence approach relies on a two-stage procedure. In the first stage, effects are tested at the individual level using a standard hypothesis-testing approach for a given significance level (${\alpha}_{individual}$). In the second stage, the proportion of observed individual-level effects (the observed *prevalence*) is tested against ${\alpha}_{individual}$, which is the type-1 error rate of the individual-level test. This is done, using a one-sided binomial test (for which a separate significance level is used, here termed ${\alpha }_{prevalence}$, which may be different from ${\alpha }_{individual}$). Due to the one-sidedness of GNT, all confidence intervals on the prevalence of effects include 100% by definition (i.e., all individuals show an effect), and the crucial aspect is whether the lower bound of the confidence interval exceeds ${\alpha }_{individual}$. Hence, a significantly higher prevalence than ${\alpha }_{individual}$ means that the *global null* hypothesis, according to which no individual shows a true effect, can be rejected.

Second, the *qualitative individual differences* approach (Haaf & Rouder, 2019; Rouder & Haaf, 2021; henceforth QUID) quantifies the relative support for the presence of “qualitative differences” in effects, that is, inter-individual differences in effect signs, by performing a Bayesian model comparison over a family of hierarchical models with different constraints (Haaf & Rouder, 2019). Specifically, QUID models RTs using a standard linear model, where individual-level effects are treated as random effects. Then, to quantify the support for an effect while allowing for qualitative individual differences, it relies on Bayes factors (BFs). The BF analysis compares evidence of a model that poses no constraints over the magnitude of individual-level effects, with a model in which all participants show no effect (see Rouder & Haaf, 2021, for further model comparisons, aimed at examining different hypotheses regarding the character of population variability in effects).

Third, Miller and Schwarz (2018) introduce a parametric and frequentist test, based on ANOVA. Specifically, their Omnibus ANOVA test (henceforth OANOVA) probes the joint null hypothesis that there are no systematic differences neither between experimental conditions across individuals, nor within individuals and across trials. Together, this is equivalent to the *global null* scenario we presented above. OANOVA relies on a trial-level ANOVA model, comparing the variability in the dependent variable which is explained by the combination of the experimental manipulation and its interaction with the participants against the variability explained solely by participants’ intercepts. Hence, OANOVA considers participants as fixed effects in the analysis, and enables detecting effects without assuming homogeneity in effect signs.

We applied the tests to our simulated data. For QUID, we used the default priors from the original publication (Rouder & Haaf, 2021). For GNT and OANOVA, we used an $\alpha$ of 0.05 to examine individual-level and group-level effects. For QUID, we considered $BF>3$ as evidence for an effect, $BF< 1/3$ as evidence for no effect (*global null*), and values between these thresholds ($1/3\le BF\le 3$) as inconclusive (Jeffreys, 1998). Reassuringly, all tests were able to differentiate between the two simulated scenarios, providing very strong evidence for an effect in the *non-directional differences* scenario, but not in the *global null* one. Specifically, according to GNT, the prevalence of effects on RT was clearly above the expected proportion of significant effects (${\alpha }_{individual}$) in the *non-directional differences* simulation (using a two-sided t-test for the individual-level test; 80% significant effects, one-sided 95% CI = [56, 100], p < .001), but this proportion was not higher than ${\alpha }_{individual}$ in the *global null* simulation (7% significant effects, one-sided 95% CI = [0, 100], p = .537). Using the QUID method, a random effects model with individual-level effects was overwhelmingly preferred in the *non-directional differences* simulation ($BF$= 9.27e+53), but a null model was preferred in the *global null* simulation ($BF$= 0.12). Similarly, the OANOVA test revealed significant results in the *non-directional differences* scenario (F(15, 2970) = 21.38, p < .001), and a non-significant effect in the *global null* simulation (F(15, 2970) = 1.30, p = .190).^2^

The simulations above demonstrate that adopting a non-directional approach, that is, an approach that takes into account the potential for opposite true effect signs among different participants, has the potential to reveal individual-level effects that would otherwise be missed due to high between-participant variability. Equipped with these validated tools, in the next section we use the QUID, GNT, and OANOVA tests to ask whether null results in the field of unconscious processing are driven by such inter-individual variability, or alternatively, whether they reflect the true absence of a causal effect.

### Re-examining unconscious effects

To examine whether inter-individual differences masked true unconscious priming effects in previously reported studies, we collected and tested data from eight studies that reported null results (Benthien & Hesselmann, 2021; Biderman & Mudrik, 2018; Faivre et al., 2014; Hurme et al., 2020; Skora et al., 2021; Stein & Peelen, 2021; Zerweck et al., 2021; Chien et al. 2022; all datasets and analysis scripts are publicly available online: https://github.com/mufcItay/NDT).

We had three inclusion criteria: first, since all probed tests require trial-level data, only open-access datasets providing such data were included. Second, the independent variable of all studies had to be manipulated within single participants. And third, at least one non-significant effect was reported in the original study. Overall, this search strategy yielded data associated with 26 null effects (see Appendix B in the Online Supplementary Material for details about all effects), 21 focusing on differences in RT and five on differences in signal detection sensitivity, *d’* (Green & Swets, 1966). We attempted to include as many datasets as possible, combining literature search, open data repositories, and calls on social media. We used the criteria set by the original authors for demonstrating unawareness (e.g., using objective and/or subjective measures of awareness), and a two-sided non-parametric sign-flipping test on the population mean for filtering out experiments that showed significant directional effects.^3^ Finally, we excluded participants with fewer than five trials per experimental condition and/or zero variance in the dependent variable (e.g., when accuracy was measured). Together, these data allowed us to reexamine null unconscious processing effects using a non-directional approach that takes into account the potential for differences in effect signs when testing for group-level effects. We accordingly asked whether true effects of unconscious processing were masked by population heterogeneity in effect signs. Importantly, while we aimed for collecting as many datasets as possible, this is not a systematic review or meta-analysis. Accordingly, we conducted searches using Google Scholar, targeting any study investigating unconscious processing, while reporting at least one null result. Accordingly, it should be noted that our sample might miss relevant datasets due to the lack of systematic approach.

To that end, the effects of interest were tested using GNT, QUID, and the OANOVA tests (see Appendix C in the Online Supplementary Material for an analysis of the significant directional effects which were excluded). GNT was applied to all 26 effects. In contrast, QUID and OANOVA were used on subsets of 20 and 21 of these effects, respectively (omitting five effects of signal detection sensitivity, *d’*, from both tests, and one additional RT interaction from the QUID analysis, as its current implementation only supports simple RT effects). All tests agreed on finding no reliable evidence for non-directional unconscious effects. According to GNT, the prevalence statistic was zero in 50% of the effects (maximal observed prevalence = 16%; see Fig. 2A)^4^, and the 95% one-sided CI included $\alpha =5$% in all of them. Hence, for all effects the prevalence of effects did not exceed the expected rate under the global null hypothesis. Similarly, for both QUID and the OANOVA tests, no single $BF$ or p-value revealed evidence for an effect (maximal $B{F}_{10}$ = 0.78 and all p-values > 0.05; see Fig. 2B, C). Notably, QUID obtained moderate evidence for the *global null* model in 70% of the cases (see Fig. 2B). The remaining effects were inconclusive. Hence, for the effects collected here, in the case of unconscious processing, the three tests revealed a highly similar pattern of results, consistent with a strong interpretation of previously reported null results as revealing the genuine absence of a causal effect of unconsciously perceived stimuli on behaviour.

Yet, the reviewed approaches also have some limitations that make it harder to draw firm conclusions based on their results. First, in contrast to frequentist tests within the Null Hypothesis Statistical Testing (NHST) tradition, QUID is designed to quantify relative evidence, and as such provides only weak^5^ control over long-term error rates (the probability of finding a false positive result or missing a true result over an infinite number of tests, with the former being more critical to our point here). Such error control promises a much-needed ‘fool-proof’ method to infer the existence of unconscious processing effects without making too many mistakes in the long run (Kelter, 2021; Lakens et al., 2020).

Second, both the model comparison approach used in QUID and the OANOVA test necessarily assume a parametric model of the data, making specific assumptions of normality and equal within-individual variance. In simulations, we find that violations of this second assumption can have dramatic effects on the specificity and sensitivity of both tests (see Appendix D in the Online Supplementary Material). This can be addressed by more complex models that are capable of handling different distribution families, but as model complexity grows, unwanted effects of assumption violations may become harder to spot and quantify. Hence, taking a non-parametric approach provides safer inferences when the form of the data-generating process is not fully known.

Lastly, since GNT is focused on the prevalence of effects, it begins with testing the significance of effects at the single subject level, thereby dichotomizing a continuous test statistic into one bit of information: significant or not. This dichotomization results in information loss and introduces an additional free parameter – the individual alpha level. This step is well justified when estimating population prevalence, but it is unnecessary for our purpose of detecting a non-directional effect at the population level. As we describe below, using a continuous participant-level statistic makes our test more sensitive (see Appendix E in the Online Supplementary Material for a direct comparison between the two approaches).

In the next section, we introduce two novel non-directional tests that take into account population heterogeneity to infer group-level effects. The tests are both frequentist and non-parametric, which addresses the above issues. Similarly to the OANOVA test, they promise a tight control for long term error-rates, but unlike it, our tests do not assume a parametric model of the data-generating process. Using a continuous within-participant summary statistic, they are also more statistically powerful than approaches that focus on a dichotomous notion of effect prevalence (see Appendix E in the Online Supplementary Material).

### Two non-parametric tests that are robust to qualitative differences

We propose two tests that follow these two principles: a within-participant effect is convincing if it is consistently evident across different trials, and if its magnitude, in standardised units, is larger than expected by chance alone.

**Fig. 3:**
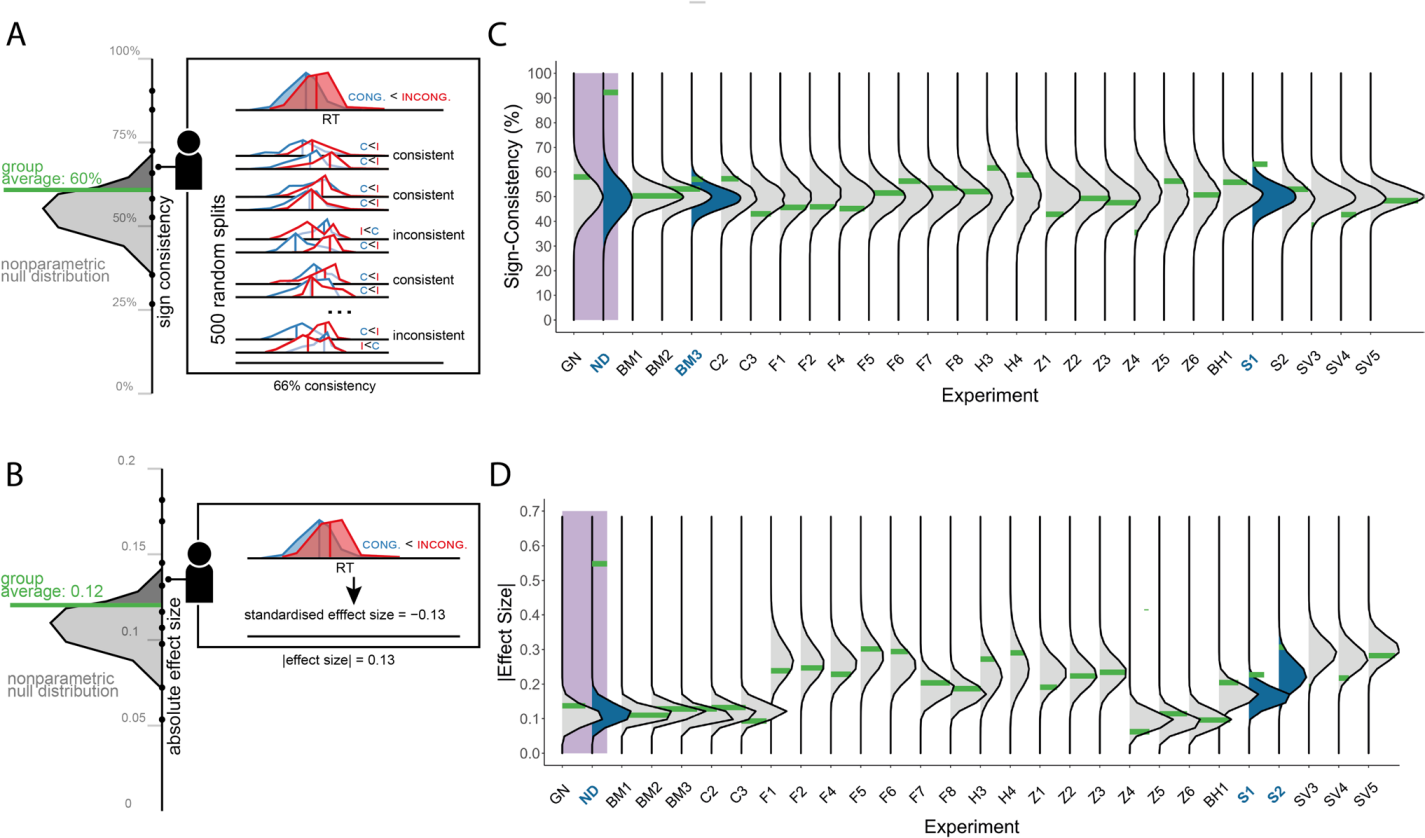
The proposed tests. Two frequentist, non-parametric, tests for non-directional effects. **Panels** **A and** **B:** Schematic illustrations of the sign consistency test (**A**) and absolute effect size (**B**) tests, using the same conventions as in Fig. 1 (C = congruent, I = incongruent). In Panel A, participant-wise sign consistency is quantified as the proportion of random splits of experimental trials, for which both halves display the same qualitative effect (C > I or I > C). The upper row illustrates the overall reaction time (RT) data for one participant, and each row below shows one split of the data. For each half we compare the mean of congruent and incongruent RT distributions, to test if the direction of the difference in the two halves is consistent or not. The averaged consistency score across participants (plotted in green) is then compared to the non-parametric null (plotted to the left of the framed box), to obtain a significance value. Panel B similarly depicts the scheme for quantifying participant-wise absolute effect size. Significance value is obtained in the same way described for the sign consistency test. In this hypothetical case, the group does not show an effect according to both tests, as the average score is well within the null distribution. Both tests can be used with other measures, such as *d’*, correlations, and indexes of metacognitive sensitivity. **Panels** **C and** **D:** The results of applying the tests to effects that produced null results in a non-parametric directional test (N = 26). **Panel C:** The results obtained by the absolute effect size test for the same datasets (N = 26). Significant results, for which the estimated group-level mean sign consistency (C) or absolute effect size (D) statistic is greater than 95% of the null distribution, are marked in blue. As in Fig. 2, the x-axis lists effect labels

First, the *Sign Consistency* test examines the consistency of effects within participants by estimating the probability that splitting the trials of an individual into two random halves would result in both halves showing the same qualitative effect (e.g., for both halves, the performance in the congruent condition is higher than in the incongruent condition; see Fig. 3A). By doing this many times (in our implementation, 500 times), we can measure how often the two halves agree. Following this strategy, we estimate the consistency of effect signs within each individual by measuring the frequency of consistent results across splits. Then, we compare the group-mean consistency score against a null distribution: 10,000 samples of group-level consistency scores, obtained after randomly shuffling the experimental condition labels within participants, effectively breaking any correlation between conditions and the dependent variable (here, to speed up the computational process, for each participant, 100 permutations were created, from which we randomly sampled a single permutation in each null distribution sample; Stelzer et al., 2013). Hence, our null distribution reflects the expected consistency of within-participant effects when the dependent variable of no single participant is sensitive to the experimental manipulation (since by shuffling the labels of the independent variable within participants we nullified its potential effect on the dependent variable).

As an alternative test, we propose the *Absolute Effect Size* test,^6^ which examines whether the absolute value of within-participant effect sizes exceeds the absolute value of such effects obtained solely due to noise (for a similar measure of consistency, see the modulation index in Buaron et al., 2020). To that end, we estimate the standardized effect size of each participant (here using Cohen’s *d* for RT effects, and *d’* for accuracy effects), and compute the group average. We then compare the group average of absolute effect sizes with a label-shuffled null distribution, using the same procedure to construct the null distribution as we do for the sign-consistency test above.

An easy-to-use implementation of both tests is available as part of the signcon R package (https://github.com/mufcItay/signcon; see Appendix F in the Online Supplementary Material for extensions of the sign-consistency test to use cases that diverge from simple mean difference between conditions. Similar extensions can be implemented for the absolute effect size test).

We applied both tests to the same effects examined in Fig. 2 (all studies for which a directional test did not produce significant results; see Appendix G in the Online Supplementary Material for an analysis of the excluded significant directional effects). The results revealed a similar picture to the one provided by the previous analyses (see Fig. 3B). First, for the simulated datasets, the sign consistency test obtained non-significant results in the *global null* scenario (SC = 58%, p = .138), and detected an effect in *non-directional differences* scenario (SC = 92%, p < .001). Similar results were obtained for the absolute effect size test (|ES| = 0.14, p = .108 and |ES| = 0.55, p < .001, respectively). Second, for the empirical datasets, the vast majority of cases did not show significant effects, with three exceptions. In two cases, the absolute effect size test revealed a non-directional effect of an unconsciously presented cue on wagering decisions (S1: |ES| = 0.23, p = .003 and S2: |ES| = 0.31, p = .027). Only the first of the two showed a non-directional effect according to the sign consistency test (S1; SC = 63%, p = .003). In a third case, the sign-consistency test, but not the absolute effect size test, revealed a scene-object congruency effect (Biderman & Mudrik, 2018; SC = 57%, p = .041). Although these effects were not detected by GNT, the prevalence of observed proportion of individual-level effects was above zero for all three (12%, 16% and 11%, respectively). Thus, despite some evidence for non-directional effects, the overall picture remained the same, hinting at minimal qualitative inter-individual differences in unconscious processing.

Together, five different analysis methods support the conclusion that by and large, unconscious priming effects are not masked by individual differences. Yet one can still claim that these statistical tests are simply not sensitive enough to detect qualitatively variable, non-directional effects, even when those exist. To test this claim, we conducted two additional analyses: First, we used simulations to estimate the sensitivity of our solutions to non-directional effects with various effect sizes, determined according to previous analyses on unconscious processing (Meyen et al., 2022) and cognitive control (Rouder et al., 2023). The results corroborated the concerns for lack of power when using common unconscious processing settings of the number of participants and trials (see Baker et al., 2021, for a detailed analysis of the contribution of both factors to power). Yet, we conducted further analysis showing that given the (low) power estimates we found, the number of significant effects obtained by the absolute effect size test would be surprisingly low if an unconscious effect existed in all or even one-eighth of the datasets (see Appendix H in the Online Supplementary Material). Second, to provide positive-control for these methods and show that they can be used to reveal such hidden effects in other fields, we collected additional, openly accessible, datasets from studies conducted in different fields of research within experimental psychology. We then used our non-parametric tests on these datasets, demonstrating its potential benefit in determining whether a null result at the group level hides true, but variable, effects at the individual participant level.

### Positive control: Testing within-participant non-directional effects across experimental psychology studies

We used the proposed tests to expose hidden effects that were not revealed by standard directional tests in various fields of research (see Appendix I in the Online Supplementary Material for the same analysis using the three other tests). To that end, we exhausted all data from different open-access databases (the Confidence Database (Rahnev et al., 2020), the Reproducibility Project (Open Science Collaboration, 2015), and the Classic Visual Search Effects open dataset (Adam et al., 2021)). We used the same inclusion criteria from the unconscious processing studies analysis, detailed above. Again, effects that were significant according to a non-parametric, directional sign-flipping test on the population mean were filtered out. Overall, we collected data associated with 136 non-significant effects (121 from the Confidence Database, four from the Reproducibility Project, eight from the Classic Visual Search Effects open dataset and three from the social media query). In all cases, participants were excluded for having fewer than five trials per experimental condition and/or zero variance in the dependent variable.

We grouped the different effects into three categories, according to research topics and the analysis we used to test them: first, we tested for effects of participants’ responses in two-alternative forced choice tasks on their confidence ratings in all datasets from the Confidence Database (Rahnev et al., 2020; retrieved on 23 January 2023), by comparing the mean confidence ratings between two different responses. Second, we used the same Confidence Database datasets to test for metacognitive sensitivity effects of response. Metacognitive sensitivity, that is, the agreement between objective accuracy and subjective confidence, was quantified as the area under the response-conditional type-2 Receiver Operating Characteristic curve (Meuwese et al., 2014 here we also excluded datasets that did not include accuracy scores; the remaining 47 effects were analyzed). Third, we grouped effects from the Reproducibility Project (Open Science Collaboration, 2015), the Classic Visual Search Effects open dataset (Adam et al., 2021), and a single study from the social media query (Battich et al., 2021) under a more general ‘Cognitive Psychology’ category. For these studies, we tested the sign consistency and significance of absolute effect size for the effect tested by the original authors (averaged difference or interaction effects).

**Fig. 4:**
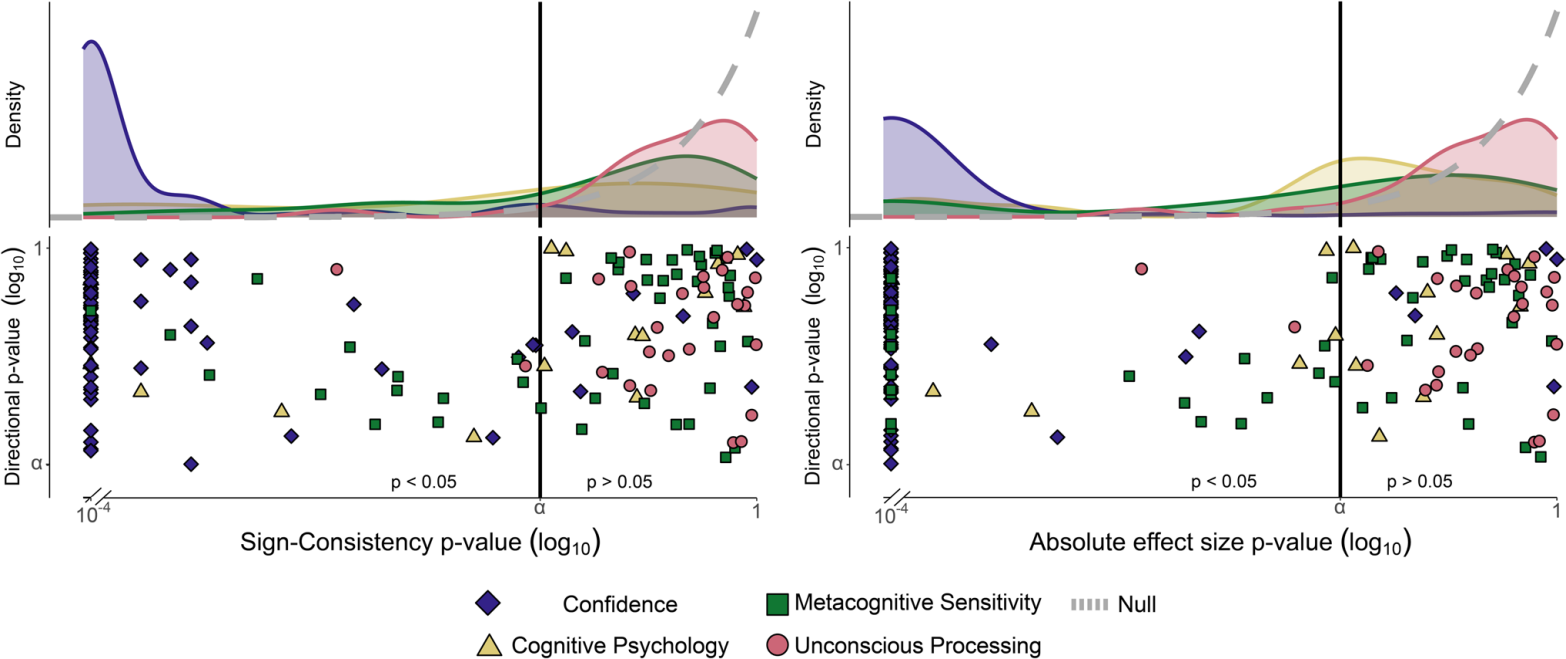
Positive control: Reanalysis of effects from other fields. The results of the sign consistency (**left**) and absolute effect size tests (**right**) for null directional effects from different cognitive psychology fields. Blue rhombuses and green squares indicate the outcomes for datasets from the Confidence Database (Rahnev et al., 2020) that were analysed to reveal differences in confidence and metacognitive sensitivity between responses, respectively. Yellow triangles indicate the outcomes for effects from various cognitive psychology studies. Finally, for comparison purposes, we also plot here in pink the results of the studies on unconscious processing (N = 26; circle markers), reported in the previous section. Finally, under the null hypothesis of no non-directional effects, the distribution of p-values should be uniform between 0 and 1. The gray dashed line indicates this expected uniform distribution of p-values (log transformed). **Lower panels:** Each point depicts the $lo{g}_{10}$ transformed p-values obtained by the tests (x-axis) and a directional sign-flipping test (y-axis; datasets were filtered to exclude significant directional effects, hence the minimal directional p-value for all datasets is $\alpha =.05$). **Upper panels:** The p-value density distributions that summarize the results in the lower panel for datasets in each field

Across the entire sample, including all analyzed effects (N = 136), most effects showed significant sign consistency (62%, N = 85) and absolute effect size effects (69%, N = 94). This trend was further explored within each category. Out of 74 null confidence effects, 67 (91%) were revealed to be non-directional effects according to the sign consistency test, and 69 (93%) according to the absolute effect size test. Out of 47 null metacognitive sensitivity effects, the two tests revealed 13 (28%) and 19 (40%) significant non-directional effects, respectively. Finally, out of 15 null effects in the cognitive psychology category, 5 (33%) and 6 (40%) of the effects were revealed to be non-directional. Both tests found significant visual search in Adam et al. (2021) and multistory integration effects in all three effects from Battich et al. (2021), while the absolute effect size test also revealed an additional non-directional effect on visual search (Forti & Humphreys, 2008), and another effect on spatial attention (Estes et al., 2008). Notably, the absolute effect-size effect revealed more significant non-directional findings in all three categories, consistent with its superior sensitivity (see sensitivity analysis in Appendices E and H in the Online Supplementary Material). Across all categories, the proportion of non-directional effects is considerably higher than the 8% of the unconscious processing effects (N = 2 according to both tests), as reported above (see Fig. 4). These results validate the potential of using non-directional tests to reveal effects on cognition and perception. In striking contrast to the absence of hidden effects in the field of unconscious processing, we found compelling evidence for pronounced inter-individual differences that mask group-level effects in other domains.

However, special care should be taken when interpreting non-directional test results, and when designing experiments targeting non-directional effects (see Box A for best-practice recommendations). Crucially, whenever some nuisance factors are counterbalanced between participants, non-directional effects cannot be uniquely attributed to the independent variable of interest. In such cases, the presence of reliable within-participant effects may reflect the opposing effects of the counterbalanced factor in each group. A case in point can be found in Battich et al. (2021), who examined the hypothesis that joint attention affects multisensory integration. Critically, this hypothesis was tested by comparing two social conditions that were counterbalanced across participants, such that for half of the participants a joint attention condition was performed before a baseline condition where participants performed the same task individually, and vice versa for the other half. As a result, contrasting the two conditions within participants is identical to contrasting early and late trials. Thus, although the interaction between social condition and multisensory integration showed significant non-directional effects (sign consistency: SC = 62%, p < .001, SC = 59%, p < .001, and SC = 67%, p < .001; absolute effect size (here using $\phi$ to estimate effect sizes on response category on the first two effects): |ES| = 0.13, p < .001, |ES| = 0.13, p < .001, and |ES| = 0.29, p = .028, for two categorical response effects and the single RT effect that showed non-directional effects paralleled with null results according to directional analysis), we cannot unambiguously interpret these results as suggesting a causal, non-directional, effect of the social manipulation. This is because, under this design, the social setting condition and the order of experimental conditions are perfectly correlated within individual participants, rendering both potential drivers behind the observed effect.

Similarly, the great majority of experiments in the Confidence Database showed significant non-directional effects of response on confidence, such that individual participants were more confident in making one response while others were more confident when making the other. Specifically, the common task used in the examined experiments involves performing a perceptual decision (e.g., discriminating whether a presented Gabor was oriented to the left or to the right by responding with specific keys), and then rating the level of confidence in the perceptual response (e.g., indicating having high confidence in their previous response that the presented Gabor was oriented to the right). Here, order effects are not a concern, as the two responses are expected to be equally distributed within a block. However, since stimulus-response mapping was not counterbalanced within participants, we are unable to tell whether these effects reflect individual differences in stimulus preferences (e.g., enhanced sensory encoding for right-tilted or left-tilted gratings among different participants) or in response biases (e.g., confidence is systematically higher after reporting a decision with the index finger). Such a response bias may reasonably emerge if, for example, both “tilted right” and “high confidence” responses are made using the right finger of the right and left hands, respectively, and if a right-finger response of the right hand primes a right-finger response of the left hand.

As a general principle, counterbalancing of confounding experimental variables can be done either between participants (e.g., using a different response-mapping for odd and even participants) or within participants (e.g., changing the response-mapping between experimental blocks for all participants). While both approaches are effective in protecting against confounding of the mean tendency of the dependent measures, only within-subject counterbalancing is effective when testing for non-directional effects. Accordingly, unless all confounding variables (e.g., condition order or response-mapping) are randomized within participants, the interpretation of non-directional effects cannot be uniquely linked to causal effects of the experimental manipulation.

Importantly, although we cannot conclusively attribute these non-directional effects to social setting versus condition order in the first example, or to response versus stimulus in the second, they both constitute examples of true effects that were masked by inter-individual differences. The absence of a directional effect in Battich et al. is indicated by the fact that on average, participants showed similar levels of multisensory integration in the first and second parts of all three experiments showing non-directional effects (${M}_{D}=0.03$, 95% CI $\left[-\text{0.02,0.09}\right]$, $t\left(48\right)=1.22$, $p=.228$, ${M}_{D}=0.03$, 95% CI $\left[-\text{0.01,0.08}\right]$, $t\left(48\right)=1.47$, $p=.149$, and ${M}_{D}=-0.02$, 95% CI $\left[-\text{0.06,0.03}\right]$, $t\left(48\right)=-0.69$, $p=.493$). In the case of confidence effects, response mapping was not counterbalanced across participants in many of the considered datasets. This way, the absence of a directional effect of response is also indicative of the absence of a directional effect of stimulus. Together, these previously hidden non-directional findings make the absence of significant non-directional effects in unconscious processing a more convincing indication of the true absence of such effects at the individual-participant level.

## Discussion

What is the scope and depth of unconscious processing? Previous claims about high-level unconscious processing effects have recently been criticized for methodological reasons (Meyen et al., 2022; Rothkirch & Hesselmann, 2017; Shanks, 2017), and for lack of replicability (Biderman & Mudrik, 2018; Hesselmann et al., 2015; Moors et al., 2016; Moors & Hesselmann, 2018; Stein et al., 2020). Here, we point out that testing for effects that are consistent across individuals may be overly conservative for the question at stake. Instead, we examined if these null results might still be underlied by an effect, yet a non-directional one. That is, we tested the hypothesis that individual differences in unconscious processing mask true unconscious effects in individual participants. Adopting a non-directional approach that is robust to inter-individual differences in effects, we used a Bayesian test (Rouder & Haaf, 2021), two frequentist tests based on prevalence assessment and ANOVA (Donhauser et al., 2018, and Miller & Schwarz, 2018, respectively), and a novel non-parametric frequentist test. We examined previously reported non-significant results (N = 26), and showed they cannot be explained by inter-individual differences in effects. All tests converged on a similar picture: besides three effects that were picked up by two of the five methods (two effects according to each test), unconscious processing effects were not masked by substantial inter-individual differences.

It is important to note that our claim here is not about the presence of individual differences in unconscious processing in general, but about the likelihood that such differences in effect signs may be responsible for null group-level findings. Indeed, previous studies revealed inter-individual differences in the magnitude of unconscious processing effects (Boy, Evans et al., 2010a; Cohen et al., 2009; Van Gaal et al., 2011). For example, Van Gaal et al. (2011) used fMRI and a meta-contrast masked arrows-priming task, to show that grey matter density is correlated with the size of unconscious motor priming effects. Yet importantly, in this experiment effects were defined according to the assumption that *incongruent* trials are performed slower than *congruent* trials (trials in which primes and targets pointed to opposing and the same direction, respectively). This assumption of group coherence in effect signs is prevalent in consciousness science, and in cognitive science more broadly, with few exceptions (e.g., see Bolger et al., 2019, for a study where the direction of face orientation effects was not assumed in advance). Here, in contrast, we asked whether relaxing the assumption of effect sign uniformity could reveal unconscious effects that remain undetected using standard directional approaches.

Overall, our non-directional tests detected an effect that was missed by a standard, directional test only in three out of 26 datasets (two according to each proposed test). However, even these effects should be examined cautiously. First, neither effect survived a correction for false discovery rate for each test among unconscious processing effects (Benjamini & Hochberg, 1995; for the two sign consistency effects: SC = 57%, uncorrected p = .041, corrected p = .530, for the third experiment in Biderman & Mudrik, 2018, and SC = 63%, uncorrected p = .003, corrected p = .078; for the absolute effect size test: for the first and second experiments in Skora et al., 2021, |ES| = 0.23 and |ES| = 0.31, uncorrected ps = .003 and .027, corrected ps = .083 and .346). Hence, these effects may reflect a type-1 error. Furthermore, the more powerful absolute effect size test did not detect the scene-object congruency effect from Biderman and Mudrik (2018) as was the case for the other three tests. Lastly, Skora and colleagues expressed concerns regarding possible contamination of their measured effect by conscious processing due to regression to the mean (Shanks, 2017), suggesting that participants’ awareness may have been underestimated. This concern was further confirmed in a reanalysis of objective measures of awareness using a test tailored to yield high power in detecting awareness (Lublinsky et al., in preparation), finding significant awareness in both of their experiments. Thus, our findings may be interpreted as suggesting no masking of unconscious processing effects by population heterogeneity.

Relatedly, our investigation aimed at unmasking non-directional unconscious processing effects. Accordingly, we did not examine whether such effects also exist in direct measures of awareness, such as the ability of participants to correctly identify the prime stimulus in a forced-choice task (Reingold & Merikle, 1988). In principle, group-level chance performance in an objective measure of awareness may reflect the joint effect of participants who systematically perform above and below chance. If so, non-directional effects in the objective measure may also go unnoticed by researchers, leading them to underestimate awareness and incorrectly infer unconscious processing.

However, unlike unconscious processing effects, where effect signs are irrelevant to the theoretical question at stake, systematic below-chance performance in objective tasks is harder to interpret. Indeed, below-chance accuracy can be equally taken as a sign of awareness, or as a sign of unawareness (see Klauer et al., 1998; Skora et al., 2023). Furthermore, the reanalysis of empirical data reported in Lublinsky and colleagues (N = 79 awareness measures; in preparation), revealed only scarce evidence for non-directional effects on awareness (as indicated above, two stark exceptions to this conclusion are the two measures used in Skora et al., 2021). Thus, while our focus here was on non-directional effects in indirect measures of consciousness, testing for the presence of such effects in direct, or objective, measures, is also of critical theoretical importance.

Notably, the homogeneity of effect signs is assumed under all common dissociation paradigms used to study unconscious processing, including the double dissociation and sensitivity paradigms which had been designed to relieve other strong assumptions underlying the study of unconscious effects (Meyen et al., 2022; Schmidt & Vorberg, 2006). Our results inform these paradigms as well as the standard paradigm which was used in the studies we reanalyzed here. Specifically, both the double and sensitivity dissociation paradigms assume a consistent direction between the effects in the direct and indirect tasks. Accordingly, the lack of evidence for reversed effects reported here supports the validity of these paradigms for demonstrating unconscious effects.

While our focus here was on unconscious processing, a non-directional analysis approach can be useful in many fields of investigation where individual differences are expected. A null finding in a standard t-test or an ANOVA may indicate the true absence of an effect or a lack of statistical power, but it may also be driven by qualitative heterogeneity in participant-level effect signs. In the field of neuroimaging, the adoption of information-based, non-directional approaches famously revealed such effects that were otherwise masked by heterogeneity in neural activation patterns and fine brain structure (Gilron et al., 2017; Ince et al., 2021, 2022; Kriegeskorte & Kievit, 2013; Norman et al., 2006). In the context of this investigation, we found considerable evidence for cases where inter-individual differences mask group-level effects. These cases carry theoretical significance both in uncovering previously missed effects, and in revealing aspects of human cognition that are subject to considerable population variability (Bolger et al., 2019; Rouder & Haaf, 2021).

Previously, Rouder and Haaf (2021) suggested that such qualitative individual differences may be expected in preference or bias-based effects (e.g., Rouder & Haaf, 2021; Schnuerch et al., 2021), but not in effects that are driven by low-level perceptual and attentional processes. Consistent with this proposal, the absence of substantial evidence for variability in effect signs in unconscious processing was paralleled with strong evidence for such qualitative inter-individual differences in subjective confidence ratings (e.g., some participants are more confident in classifying a grating as oriented to the right, while others show the opposite preference).^7^ However, robust participant-level effects were masked by qualitative individual differences in other domains too, not all of them relate to higher-level preferences or biases. For example, non-directional effects of distractor presence were found in visual search experiments (Adam et al., 2021; SC > 62%, p $<$.020, for two out of eight measured effects, only one of them was significant according to the absolute effect size test: |ES| = 0.12, p < .001). Moreover, the absolute effect size test (but not the sign consistency test) revealed significant effects in two studies from the Reproducibility Project: words associated with locations had a non-directional effect on spatial attention (|ES| = 0.28, p = .041; Estes et al., 2008), and visual search was non-directionally affected by the interaction between target location and viewpoint prototypicality (|ES| = 0.13, p = .047; Forti & Humphreys, 2008). These findings echo the non-directional effects of distractor-target compatibility on action planning, previously revealed by Miller and Schwarz (2018) using their OANOVA test on data from Machado (2007, 2009). When re-analysing Machado’s data using our non-parametric tests, we find similar results: null directional effects, accompanied by significant non-directional effects (sign consistency: SC = 78%, p < .001 and absolute effect size: |ES| = 0.30, p < .001, for a target-distractor SOA of 350ms in Machado et al., 2007; sign consistency: SC = 63%, p = .025 and absolute effect size: |ES| = 0.31, p = .001, for an SOA of 650 ms in Machado et al., 2009). Thus, aside from shedding light on previous non-significant results, our preliminary findings inform previous claims regarding the plausibility of population heterogeneity in effect signs in perceptual and attentional effects in general, providing some indication that such effects may be more prevalent than previously assumed.

To facilitate the adoption of this non-directional approach in experimental psychology, we release with this paper an R package with a simple-to-use implementation of our error-controlled and non-parametric sign consistency test (https://github.com/mufcItay/signcon). We note that unlike directional tests, the validity of non-directional tests depends on counterbalancing of confounding variables not only across participants, but also across trials within a single participant. We recommend using these tests to complement standard, directional tests, taking into account the effect of additional tests on the family-wise error rate. Furthermore, although the test revealed effects in various domains, special attention should be given to statistical power when collecting data for a non-directional test, considering both the number of participants and the number of trials per participant. This is especially important when the effect size of interest is small, as is clearly the case in unconscious processing studies (more generally, when the relation between true variability between participants and measurement error is small; see Rouder et al., 2023, and Appendix H in the Online Supplementary Material). Given proper use, the test should be particularly useful in interpreting null findings at the group level (see Box A for a more detailed description of best-practice recommendations for non-directional testing). This seems highly relevant to the field of unconscious processing, where null results are becoming more prevalent, and carry theoretical significance as hinting at possible functional roles for conscious processing.

## Conclusions

Experimental demonstrations of unconscious processing have been reported for nearly 150 years now (e.g., Peirce & Jastrow, 1884), yet their reliability and robustness have repeatedly been put into question (e.g., Holender, 1986; Shanks, 2017). Here, we examined the possibility that some of the findings against such processing, reporting null results, might hide effects at the individual level, yet in opposing directions. We employed five non-directional tests to re-examine 26 null effects. Our findings suggest no role for individual differences in explaining non-significant effects at the group level. Furthermore, by expanding our exploration outside the domain of unconscious processing, we found compelling evidence for effects that were shadowed by individual differences in effect signs, nuancing views about the universality of cognitive and perceptual effects. We provide a user-friendly implementation of the non-directional tests, and recommend their use for interpreting null results.

## Supplementary Information

Below is the link to the electronic supplementary material.

Supplementary file1 (DOCX 4528 KB)

## Data Availability

All data except for the datasets from Machado et al. (2007, 2009), for which participants did not consent to having their data shared online are available at https://github.com/mufcItay/NDT

## Footnotes

1 Similar results were obtained with more realistic Wald RT distributions as detailed in Appendix A in the Online Supplementary Material.

2 Note that the unusually high degrees of freedom of OANOVA stems from modelling participants as fixed rather than random effects, as the global null model assumes no variability in effect sizes between participants.

3 Filtering out significant directional effects was done to validate previously reported null results, and to select the non-significant effects from studies that reported both significant and non-significant results. Across all RT effects, our analysis used raw RT scores, and thus our results diverged from the original results when log transformations were used (see the notes column in Appendix B in the Online Supplementary Material for details).

4 Effect labels abbreviations (sorted alphabetically): BH = Benthien and Hesselmann (2021), BM = Biderman and Mudrik (2018), C = Chien et al. (2022), F = Faivre et al. (2014), H = Hurme et al. (2020), S = Skora et al. (2021), SV = Stein and Peelen (2021), Z = Zerweck et al. (2021). For all labels, numbers denote effect indices within each study (see Appendix B in the Online Supplementary Material for the full mapping between labels and effects).

5 Specifically, p($B{F}_{10}>x|{H}_{0}$) is never higher than $1/x$. Proof: assume that $B{F}_{10}>3$ for observed values $v\in V$. That is, for every $v\in V$, $p\left(v|{H}_{1}\right)>3p\left(v|{H}_{0}\right)$. The probability of making a type-1 error $\alpha$ equals $p\left(v\in V|{H}_{0}\right)$. It follows that $p\left(v\in V|{H}_{1}\right)>3\alpha$. Since this probability cannot be higher than 1, $\alpha$ cannot exceed $1/3$ (for a similar derivation, see the universal bound in Royall, 2000).

6 We would like to thank Sascha Meyen, a reviewer of this paper, for the extremely helpful suggestion to implement and use the absolute effect size test.

7 As we note above, since in most of these studies responses and stimuli are closely correlated, these effects cannot be unambiguously attributed to stimulus preferences or response priming effects. Relatedly, more recent work reveals that such inter-individual differences in preference for specific responses or stimuli can be traced back to heterogeneity in sensory encoding Rahnev (2021).

## Code availability

All analysis code are available at https://github.com/mufcItay/NDT, using R (Version 4.3.2; R Core Team, 2023) and the R-packages *BayesFactor* (Version 0.9.12.4.4; Morey & Rouder, 2015), *coda* (Version 0.19.4; Plummer et al., 2006), *data.table* (Version 1.14.8; Dowle & Srinivasan, 2023), *dplyr* (Version 1.1.2; Wickham, François et al., 2023a), *extraDistr* (Version 1.9.1; Wolodzko, 2020), *foreach* (Version 1.5.2; Weston, 2022), *ggh4x* (Version 0.2.5; van den Brand, 2023), *ggplot2* (Version 3.4.2; Wickham, 2016), *ggpubr* (Version 0.6.0; Kassambara, 2023), *ggridges* (Version 0.5.4; Wilke, 2022), *ggtext* (Version 0.1.2; Wilke & Wiernik, 2022), *gridExtra* (Version 2.3; Auguie, 2017), *groundhog* (Version 3.1.2; Simonsohn & Gruson, 2023), *MASS* (Version 7.3.60; Venables & Ripley, 2002), *Matrix* (Version 1.6.0; Bates et al., 2010), *MCMCpack* (Version 1.6.3; Martin et al., 2011), *nleqslv* (Version 3.3.4; Hasselman, 2023), *papaja* (Version 0.1.2; Aust & Barth, 2023), *patchwork* (Version 1.1.2; Pedersen, 2022), *pracma* (Version 2.4.2; Borchers, 2022), *scales* (Version 1.2.1; Wickham et al., 2022), *signcon* (Version 0.1.0; Yaron & Mazor, 2024), *stringr* (Version 1.5.0; Wickham, 2022), *tidyr* (Version 1.3.0; Wickham, Vaughan et al., 2023b), *tinylabels* (Version 0.2.4; Barth, 2023), and *xfun* (Version 0.39; Xie, 2023).
