# Supplementary material for: Individual differences do not mask effects of unconscious processing
Source: Psychon Bull Rev. 2025 Mar 24;32(5):1969–86. doi: 10.3758/s13423-025-02679-5 (PMC12425849; doi:10.3758/s13423-025-02679-5)
Supplement: Supplementary file 1 — Supplementary file1 (DOCX 4528 KB) [file 13423_2025_2679_MOESM1_ESM.docx]

**Supplementary Materials**

# Appendix A. Simulating non-directional unconscious processing effects with Wald distributions

To complement the simulation of normally distributed RTs reported in the main text (under the section titled ‘Simulating non-directional unconscious effects’), we ran an additional simulation using more realistic Wald distribution. Importantly, unlike normal distributions and similarly to RT distributions, Wald distributions are strictly positive and right-skewed. Again, we simulated a *non-directional differences* scenario, and a *global null* scenario, which differed by the within-participant shape parameter $\lambda$ ($\lambda$=101250 and $\lambda$=9112.5 in the former and later scenarios, respectively). Accordingly, RTs were sampled from shifted Wald (SW) distributions ($RT_{i,c}\sim\mathcal{SW}\left( \mu+c*e_{i},\lambda,\tau\right)$, where $\mu$ was set to 450ms and $\tau$ was set to 200ms, to mimic typical RT distributions).

First, as in the original analysis, a t-test did not find an effect in neither scenario (*non-directional differences*: $M=5.03$, 95% CI $\left[ -5.16,15.21 \right]$, $t\left( 14 \right)=1.06$, $p=.308$; *global null*: $M=0.48$, 95% CI $\left[ -7.79,8.75 \right]$, $t\left( 14 \right)=0.12$, $p=.903$). More crucially, as can be seen in Table A1, the results of all probed tests were similar to those obtained with normally distributed RTs, finding evidence for an effects only in the non-directional scenario.

*Table A1. Resutls for simulated data under the non-directional effect and global null scenarios using Wald distributions*

| Test | Non-directional effects scenario | Global Null scenario |
| --- | --- | --- |
| GNT | 73% significant effects, p < .001 | 0% significant effects, p > .999 |
| QUID | BF = 2.43e+46 | BF = 0.29 |
| OANOVA | F(15,2970) = 18.64, p < .001 | F(15,2970) = 1.06, p= .386 |
| Sign-Consistency | SC = 91%, p < .001 | SC = 53%, p= .327 |
| Absolute Effect Size | \|ES\| = 0.52, p < .001 | \|ES\| = 0.12, p= .438 |

# Appendix B

*Unconscious processing effects metadata*

| Study | Labels | Topic | Paradigm | DV | Notes |
| --- | --- | --- | --- | --- | --- |
| Biderman & Mudrik, 2018 | BM1-3 | Scene congruency | Masking | RT | Replication study. For all experiments, log(RT) was used in the original analysis |
| Faivre et al., 2014 | F1-8 | Multisensory integration | Masking | RT | Four experiments, with two effects in each experiment (identical/different targets). For all experiments, log(RT) was used in the original analysis |
| Stein & Peelen, 2021 | SVP1-5 | Location effects + PAS (detection) | CFS | d’ | Two experiments (3 and 4 in the paper), measuring effects in different prime-mask SOAs |
| Zerweck et al., 2021 | Z1-7 | Numerical Priming | Masking | RT | Two experiments (2 and 3 in the original paper), measuring effects in different SOA / Contrast conditions |
| Benthien & Hesselmann, 2021 | BH1 | Numerical Priming | CFS | RT | Interaction effect - prime congruency X location certainty |
| Hurme et al., 2020 | H1-4 | Colours | TMS + Metacontrast Masking | RT | Redundant target effect (TMS / Masking X Blue / Red) |
| Skora et al., 2021 | S1-2 | Instrumental Learning | Masking | d’ | Regression to the mean as a confound according to authors |
| Chien et al., 2022 | C1-3 | Semantic priming | CFS | RT | Word, Picture, and trait discrimination tasks |

# Appendix C. GNT, QUID and OANOVA results for datasets showing a directional effect


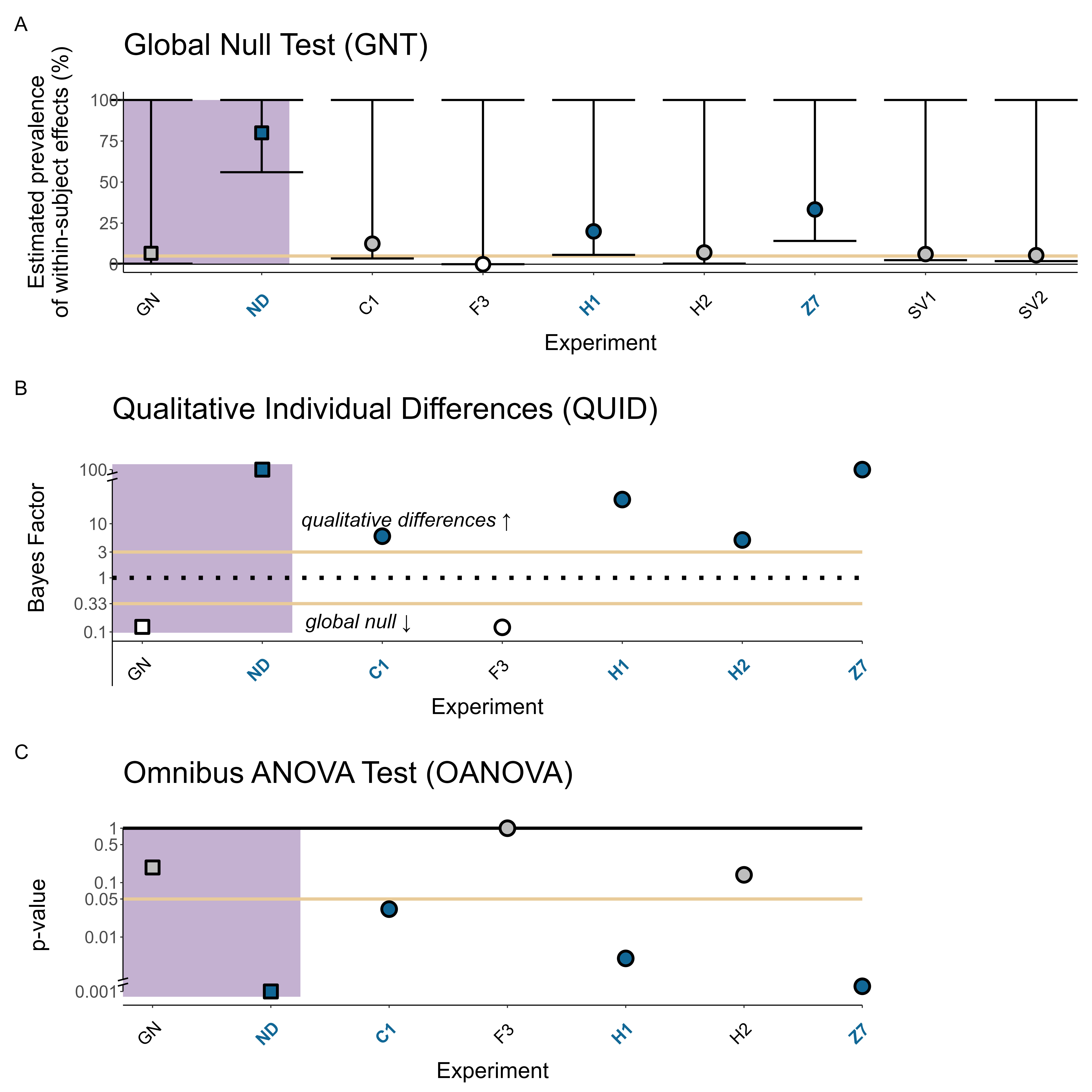


*Note*. The results of applying the GNT (A), QUID (B) and OANOVA (C) tests to effects that produced significant results in a non-parametric directional test. Same conventions as Fig. 2.

# Appendix D. Violating the equal within-individuals variance assumption

We used the simulation scheme described in the main text (see section ‘Simulating non-directional unconscious effects’), to test the consequences of violating the equal within-individuals variance assumption for both QUID and the OANOVA test. We compared the distribution of Bayes factors and p-values obtained by applying QUID and the OANOVA test to generated data meeting and violating the equal within-participants variance assumption. In the first, equal-variance case, the within-individual standard deviation was low ($\sigma_{w}=10$) for all participants. In the second, unequal-variance case, the within-individual standard deviation was low ($\sigma_{w}=10$) for all participants except one, for whom it was set to a high value ($\sigma_{w}=50$). As in the main simulation, the effect sizes of each participant were sampled from a normal distribution centred at zero ($e_{i}\sim\mathcal{N}\left( 0,\sigma_{b} \right)$; where $e_{i}$ denotes the effect size of the $i^{th}$ participant). Within this framework, we examined two scenarios: a *non-directional differences* scenario where participants are differentially affected by the experimental manipulation ($N_{p}=20$, $\sigma_{b}=2$), and a *global null* condition where all participants are unaffected by the experimental manipulation ($N_{p}=20$, $\sigma_{b}=0$). In both scenarios, we simulated random data in 500 iterations, and used the same number of trials per condition (the total number of trials, $N_{t}=200$).

To examine the tests’ specificity, we measured the proportion of iterations where evidence for an effect was erroneously found in the global null condition. In the equal-variance case, all iterations provided evidence for the lack of an effect according to QUID (all BFs < $\frac{1}{3}$). Similarly, non-significant results were found by the OANOVA test in 95% of the iterations. However, in the unequal-variance case, false-positives were obtained in 8% of the QUID Bayes Factors (BF > 3), and 7% showed inconclusive evidence. Again, the OANOVA test showed a similar pattern, detecting falsely significant effects in 17% of the iterations. Thus, we show that the specificity of these tests is compromised by violations of the equal-variance assumption.

We then analyzed the tests’ outcomes in the non-directional differences scenario to examine their sensitivity. When the equal-variance assumption was met, both tests found evidence for an effect (all BFs > 3, and all p-values < 0.05). In contrast, in the unequal-variance case, only 32% of QUIDs BFs showed evidence for an effect, whilst 40% showed evidence for no effect (the remaining 29% were inconclusive). Similarly, the OANOVA test found significant effects in only 57% of the iterations. Hence, both tests missed true effects when the assumption was not met, demonstrating that their sensitivity is compromised by violations of the equal-variance assumption.

# Appendix E. Comparing the power of the GNT and the proposed tests

To examine the sensitivity of GNT and compare it with both the sign consistency and absolute effect size tests, we conducted a power analysis, simulating two scenarios under the simulations scheme described in the main text (see section ‘Simulating non-directional unconscious effects’): First, a *non-directional differences* scenario where an effect exists for each participant but it is inconsistent within participants ($e_{i}\sim\mathcal{N}\left( 0,\sigma_{b} \right)$; where $\sigma_{b}$=1.5). Second, a *directional effect* scenario, with individual variation around a positive mean effect size ($e_{i}\sim\mathcal{N}\left( 1,\sigma_{b} \right)$; where $\sigma_{b}$=1.5). For each scenario, we manipulated the number of simulated participants ($N_{p}$=10/30/50) and trials ($N_{t}$=50/100/500) across 1000 random iterations, with the within-participant SD ($\sigma_{w}$) set to 10 in both scenarios. Statistical power was defined as the proportion of significant results for each test ($\alpha=.05$). While all tests were similarly sensitive when applied to well-powered datasets (e.g., when $N_{p}$=50 or $N_{t}$=500), both the sign consistency test, and yet more so, the absolute effect size test proved to be more sensitive in the remaining conditions (see Baker et al., 2021 for a more comprehensive power analysis of a directional test in the *directional effect* scenario).

**Figure E1**

*Comparing the Sensitivity of the Proposed Tests and GNT*


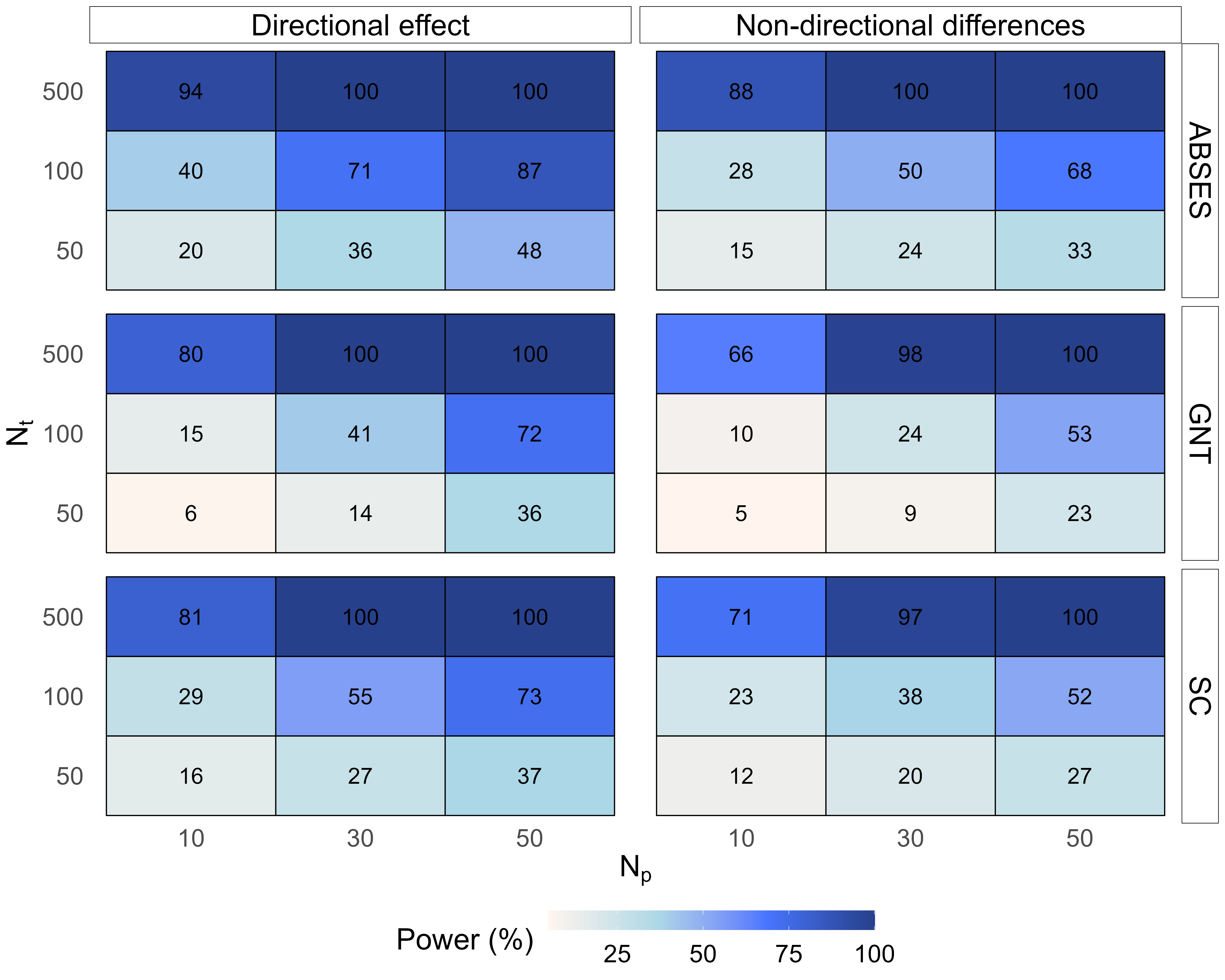


*Note*. Comparing the sensitivity of the proposed tests and GNT. Power analysis for the absolute effect size (ABSES), sign consistency (SC) and the global null (GNT) tests for simulated datasets. Top panel: Each cell depicts the percent of iterations where ABSES, SC and GNT resulted in significant effects (upper and lower panels, respectively). Rows and columns correspond to the number of simulated participants ($N_{p}$), and the number of simulated trials per participant ($N_{t}$), respectively. Left panel: the results of the ABSES, GNT and SC in the *directional effect* scenario. Right: the results of the ABSES, GNT and SC in the *non-directional differences* scenario. The number in each cell denotes the % of significant effects (power) in each simulated condition (across 1000 iterations), and darker blue colors indicate higher power.

# Appendix F. Extending the sign consistency test to additional use cases

We provide two code examples to demonstrate how to extend the sign-consistency test implemented in the signcon R package (<https://github.com/mufcItay/signcon>) to common use case: F.1. A 2 X 2 interaction, and F.2. calculating SDT’s d’. For both examples we will use simulated trial-level data, mimicking a 2X2 within-participant design.

## idv iv iv2 dv_response dv_RT
## 1 1 0 0 0 517.5380
## 2 1 0 1 0 553.3246
## 3 1 0 0 0 485.9156
## 4 1 0 1 0 482.6025
## 5 1 0 0 0 462.7242
## 6 1 0 1 0 589.1383

## Appendix F.1: 2X2 interction effect:

To test for an interaction effect we override the default test-consistency summary function. In this example, we use a function that summarizes RTs (‘dv_RT’) by calculating the mean difference between the two conditions of the second variable (‘iv2’). This summary function will be applied to the dependent variable (‘dv_RT’) of different splits of the data, under each condition of the independent variable (‘iv’), when calculate sign-consistency scores per participant.

# the interaction summary function for an interaction effect
interaction_summary_function <- function(data) {
 if(length(unique(data$iv2)) != 2) {
 return(NA)
 }
 # we use mean to summarize the RT under each level of 'iv2'
 res <- mean(data[data$iv2 == 1,]$dv_RT) -
 mean(data[data$iv2 == 0,]$dv_RT)
 return(res)
}
# run the sign-consistency test
sc_interaction <- test_sign_consistency(data, idv = 'idv', iv = 'iv', dv = c('iv2', 'dv_RT'), summary_function = interaction_summary_function)

## Appendix F.2: d’ effect:

To test for an effect on sensitivity (d’) we override the default test-consistency summary function, to compute the normalized rate of responses given for a reference stimulus (here, the reference stimulus is encoded as ‘1’). As explained above, since this summary function is applied to each condition and participant under the independent variable (‘iv’) when calculating sign consistency scores, the sign-consistency test would test for consistent d’ sign between different splits of the data for the respective participant.

# since in this use case there is only one dependent variable, the 'data' argument
# is a vector containing all of the dv_response values for the sampled split
dprime_summary_function <- function(data) {
 # count how many '1' responses were given
 cnt <- sum(data)
 # get the total number of trials in this split
 len <- length(data)
 # correction for edge cases where participants only give one response (0 / 1)
 floor_rate <- 1/(2*len)
 ceiling_rate <- 1 - 1/(2*len)
 # calculate the observed rate of 1 responses
 rate <- ifelse(cnt == 0, floor_rate,
 ifelse(cnt == len, ceiling_rate,
 cnt / len))
 return (qnorm(rate))
}
# run the sign-consistency test
sc_dprime <- test_sign_consistency(data, idv = 'idv', iv = 'iv', dv = 'dv_response', summary_function = dprime_summary_function)

# Appendix G. Results of the novel non-parametric tests for datasets showing a directional effect

**Figure G1**

*Analysis of datasets showing a directional effect*






*Note*. The results of applying the proposed tests for datasets showing a directional effect (N = 7), using the Sign Consistency test (upper panel) and the Absolute Effect Size test (lower panel). The x-axis lists effect labels. Same conventions as Fig. 3.

# Appendix H. Empirically informed power estimation for the novel non-parametric tests

To examine whether lack of power can explain not finding convincing evidence for non-directional unconscious processing effects (Fig. 3), we examined the sensitivity of both the sign-consistency and absolute effect size tests in detecting effects of empirically relevant studies. To that end, we simulated *non-directional differences* scenarios with different degrees of true between participants variance ($\sigma_{b}\in\{1,1.5,2\}$) and fixed amount of within participant variability ($\sigma_{w}=10$). These parameters were chosen based on previous works where the ratio $\frac{\sigma_{b}}{\sigma_{w}}$ was estimated to values ranging between .04 and .15 (M = .1, SD = .04) in six unconscious processing datasets (Meyen et al., 2022), while in another work estimating the same parameter in 24 cognitive control studies (Rouder et al., 2023; where no unconscious manipulation was used) values ranged between .05 and .36 (M = .14, SD = .08). Then, we determined the number of trials and participants in the simulated datasets according to the parameters used in the unconscious processing datasets we obtained by calculating the 25%, 50%, and 75% percentile of both parameters. This resulted in additional simulation conditions where the number of participants was set to $N_{p}\in\{17,22,34\}$ and the total number of trials was set to $N_{t}\in\{48,144,208\}$. All other simulation parameters were the same as detailed in Appendix E.

From the results of this power simulation we obtained an estimate for the power of both the sign consistency and absolute effect size tests^[[1]](#footnote-2)^, in common sample size settings, for an effect of interest of $\frac{\sigma_{b}}{\sigma_{w}}=.15$. We then used a prevalence test to test whether the obtained power estimate is compatible with the low observed prevalence of unconscious processing effects we found. Due to the concern regarding contamination by conscious processing in the two effects from Skora et al., 2021 (see the Discussion for more details), we excluded these effects from this analysis, resulting in an observed prevalence of zero out of 24 tested effects (i.e., no non-directional unconscious effects were found). Accordingly, we tested if finding no unconscious effects is surprising relative to the expected prevalence according to our power estimate (here using the power estimate of the absolute effect size test which was highest amongst the two probed tests (61%), and assuming that all studies had true non-directional effects). Indeed, this was the case when comparing the observed rate of significant effects with the expected rate if all, or even one-eighth of the tested contrasts had true non-directional effects (N = 3 out of 24 tested effects), yet were not detected due to a lack of power ($CI_{95}$ = [0, 12], p < .001, and p = .047, respectively). Furthermore, even when including the two arguably conscious effects found by Skora and colleagues in the analysis, the prevalence of observed effects remained significantly lower than expected if a third of the effects were true (p = .037; using as the observed prevalence of effects across all datasets, 8%). Hence, we interpret these results as suggesting that for an effect size of $\frac{\sigma_{b}}{\sigma_{w}}=.15$, the results are unlikely to be explained simply by lack of power.

**Figure H1**

*Data Informed Power Analysis of the proposed tests*


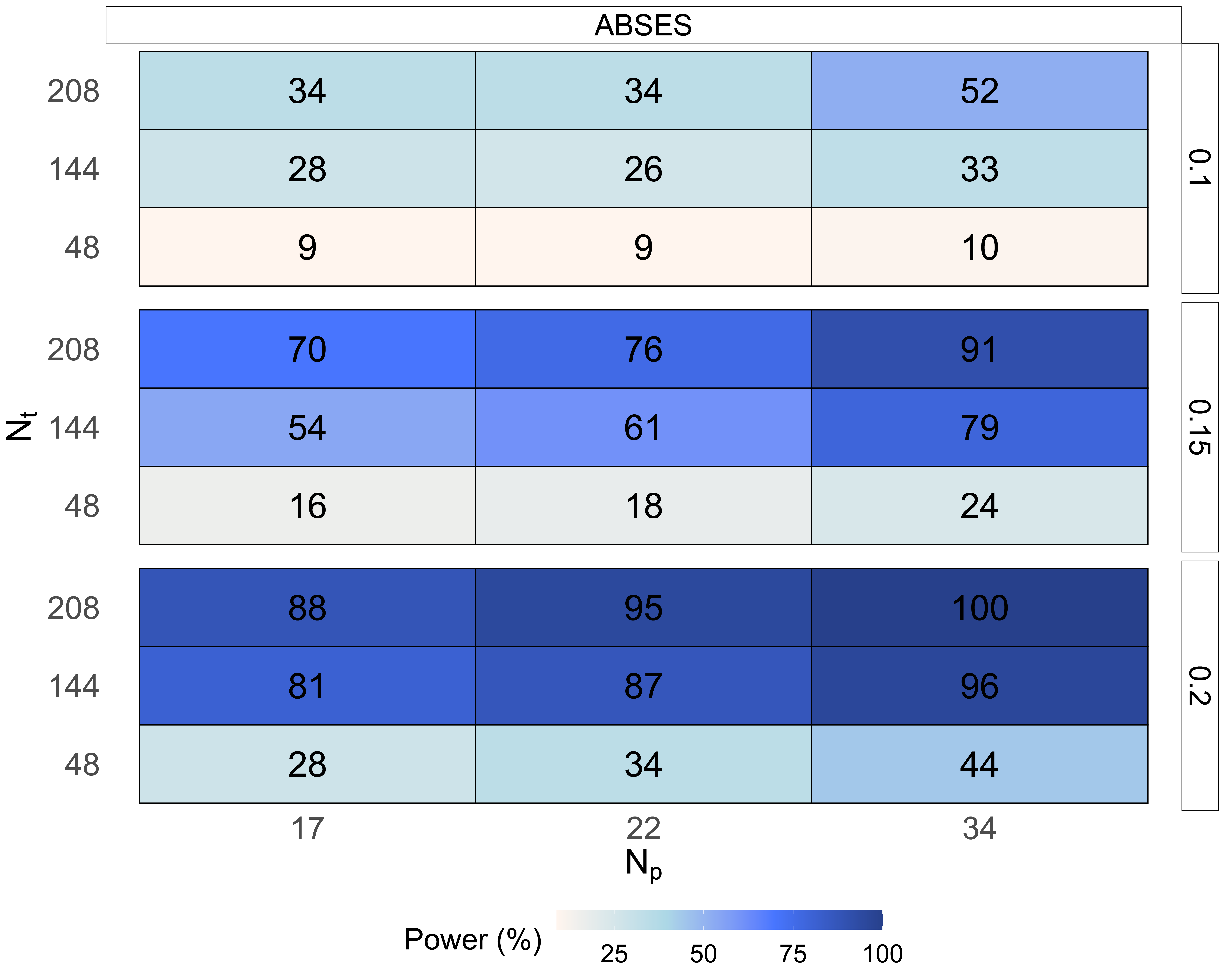


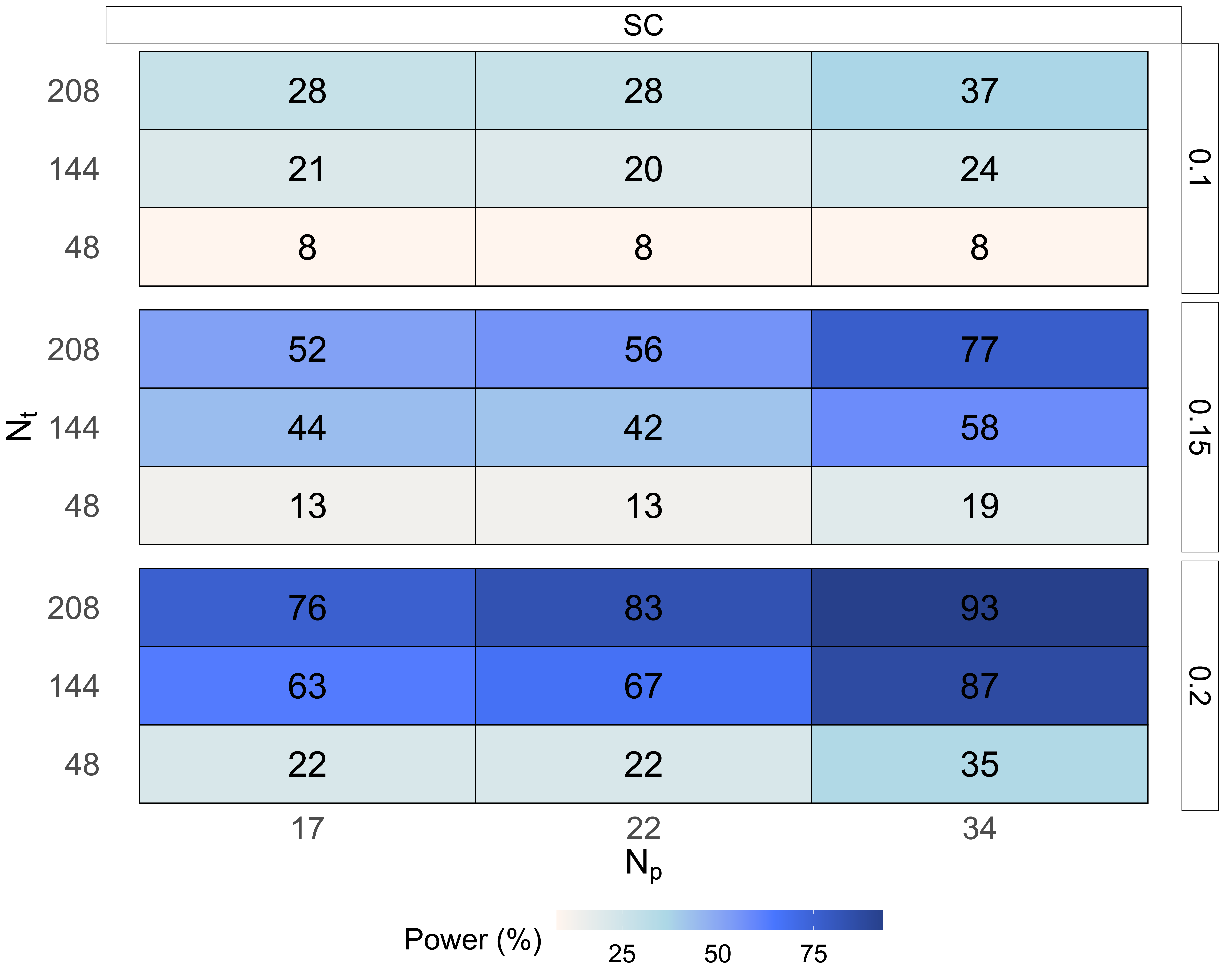


*Note*. Power analysis for various *non-directional differences* scenarios. The results for the sign consistency (SC) and the absolute effect size (ABSES) tests are presented on the upper and lower plots, respectively. Each panel depicts the results for different effect sizes ($\frac{\sigma_{b}}{\sigma_{w}}\in\{.1,.15,.2\}$). Within each panel, the x and y axes depict different settings for the number of participants ($N_{p}$) and the total number of trials across two conditions ($N_{t}$), both determined according to the 25, 50 and 75 percentiles of these parameters in the unconscious processing datasets we collected. The number in each cell denotes the % of significant effects (power) in each simulated condition (across 250 iterations), and darker blue colors indicate higher power.

# Appendix I. Testing for effects from other domains with the alternative tests

For completeness, we report here the results of the GNT, QUID, and OANOVA tests for all datasets outside the domain of unconscious processing reported on the main text, which were non-significant effects according to a directional test^[[2]](#footnote-3)^ (see section ‘Positive control: Testing within-participant non-directional effects across experimental psychology studies’ for the same analysis using the proposed tests). Crucially, this analysis should be interpreted with caution given the results we report in Appendix D and E, showing potential issues with the sensitivity and/or specificity of these tests.

Overall the results were similar to the one found with the proposed tests: First, the vast majority of datasets from the confidence database showed non-directional effects (93% and 96%, for GNT and OANOVA, respectively). Similarly, GNT found metacognitive sensitivity effects in 28% of the datasets. Lastly, within the ‘Cognitive Psychology’ datasets category, both GNT and OANOVA were significant for all three effects from Battich et al. (2021) on multisensory integration (since all of these effects involve interactions, they were not analyzed using QUID), while obtaining significant results for only one of the two visual-search sign consistency effects by the absolute effect size, OANOVA and QUID tests, or none of them for GNT, and a significant effect in (Estes et al., 2008) according to both OANOVA and GNT in agreement with the significant absolute effect size result for this effect (overall cognitive psychology datasets, GNT, QUID and OANOVA found 20%, 10%, and 29% of significant effects, out of 15, 10, and 14 effects examined by each test).

**Figure I1**

*The Results of the Alternative Tests on Effects From Other Fields*


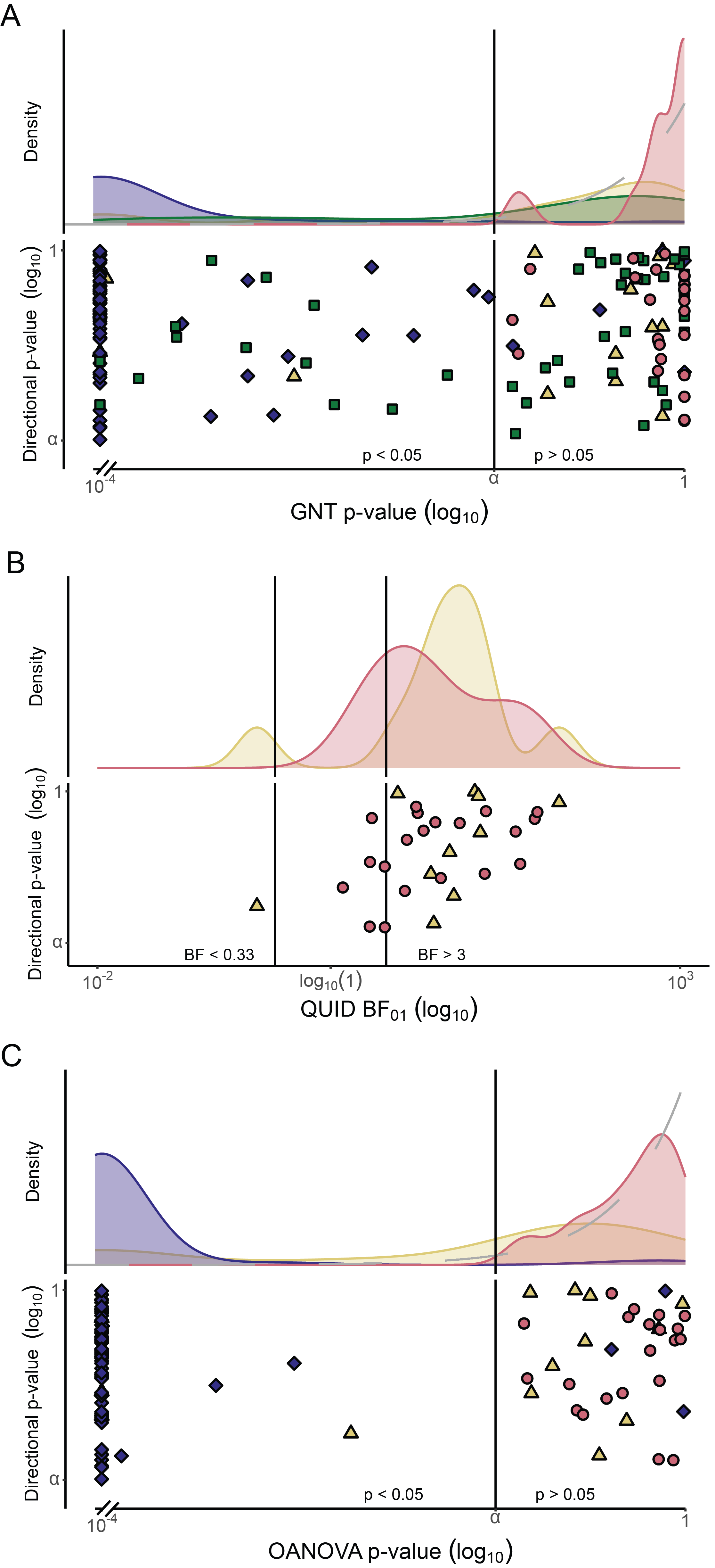


*Note:* The results of applying the GNT (A) (N=162), QUID (B) (N=30), and OANOVA (C) (N = 103) tests to null directional effects from different cognitive psychology fields. Same conventions are used as in Figure 4 in the main text. Effects that are incompatible with OANOVA or QUID were excluded from this analysis. In panel B the two black vertical indicate BF criteria of 3.

#

# BOX A: Non-directional testing: best practice recommendations

- **When should we use the non-directional approach?**
  - Not all hypotheses are suitable for examination under the non-directional approach. Since the non-directional approach is targeted at detecting the presence of effects rather than their direction, it cannot be used to establish average differences between conditions at the group level (e.g., when comparing memory performance for items presented first and later in an experiment, rejecting a non-directional hypothesis does not entail evidence for an overall primacy or recency effect on recollection). In the case of unconscious processing, the theoretical question is regarding the presence or absence of a difference between the two conditions at the single-participant level, and as such, it lends itself to non-directional testing. Thus, selecting whether to use the non-directional or directional approach is directly linked to the theoretical question at stake.
  - A general recommendation is to plot the data of individual participants with a measure of within-participant variability across trials (e.g., within-participant CI). As can be seen in Figure 1, the individual-level confidence intervals may guide researchers regarding the plausibility of non-significant group-level effects resulting from population variability in effect signs.
- **Which test should be used?**
  - When testing whether the experimental manipulation affects the dependent variable (e.g., either main or interaction effects on reaction times, accuracy, brain activity etc.), unless normality and equal variance of within-participant variability can be assumed with high certainty, we recommend using the absolute effect size test due to is computational efficiency and superior sensitivity.
  - When these assumptions hold, QUID or OANOVA can be used for effects that are measured on a trial-by-trial basis (as opposed to effects measured by summarizing data from multiple trials, e.g., d’ or correlation effects). Specifically, when prior data is available, we advise incorporating it into the analysis using QUID, and when examining an interaction effect, OANOVA provides an easy-to-use solution.
  - To test for the prevalence of individual-level effects, rather than the mere existence of an effect at the group level, we recommend using the prevalence approach (Ince et al., 2021, 2022). More specifically, we recommend using GNT (Donhauser et al., 2018) to test whether the data provide evidence for the presence of an effect for at least a single individual.
- **Non-directional tests require within-participant counterbalancing of confounding variables**
  - As we discuss in the text, special care should be given to counterbalancing of confounding variables when using the non-directional approach. Specifically, unlike standard directional tests, the effects of confounders are not averaged out at the group level when counterbalanced across participants. Thus, when designing an experiment to reveal non-directional effects, counterbalancing should be done not only across participants but also across trials within participants. It should be noted however that within-subject counterbalancing can result in a more confusing task from the participants’ point of view, requiring researchers to balance design simplicity with inferential scope.
- **How to interpret non-directional effects?**
  - In contrast to directional tests, where signal is measured relative to variability across individuals, in non-directional tests it is measured relative to variability across different trials, within an individual. Hence, a positive result of a directional test indicates that effects are consistent between participants, while a non-directional test reveals the presence of an effect on the dependent variable within participants, regardless of the alignment of within participants effects across participants.
  - A significant non-directional effect without a corresponding directional effect suggests reliable variability in effect signs across individuals. Whether this variability reflects transient or stable individual differences can be further tested by correlating individual effect scores from two experimental sessions: stable differences should result in a positive correlation. Whenever stable individual differences are observed, further research may be needed to identify the relevant personal traits that interact with the experimental manipulation.

1. Importantly, this decision incorporates the fact that the proposed tests may also detect small directional effects that remain undetected using standard tests, due to heterogeneity in individual-level effects. In this case, estimating the power of the test based solely on the ratio of between and within participant variability might underestimate the true power of the test for the obtained datasets. Hence, we used the same effect size as was done in Meyen et al., (2022), who used it as part of a “benefit of a doubt” approach for a different set of analyses on unconscious processing effects. [↑](#footnote-ref-2)
2. Notably, since both QUID and OANOVA were developed for continuous dependent variables, with the default prior settings of QUID were set according to expected patterns for RTs, we did not use either test to analyze metacognitive sensitivity effects, and also excluded confidence effects from the QUID analysis. Similarly, QUID was not used for analyzing interaction effects, because its current implementation does not allow for such analysis. [↑](#footnote-ref-3)
